# Role of stress-inducible protein-1 in recruitment of bone marrow derived cells into the ischemic brains

**DOI:** 10.1002/emmm.201202258

**Published:** 2013-07-08

**Authors:** Shin-Da Lee, Ted Weita Lai, Shinn-Zong Lin, Chen-Huan Lin, Yung-Hsiang Hsu, Chi-Yuan Li, Hsiao-Jung Wang, Wei Lee, Ching-Yuan Su, Yung-Luen Yu, Woei-Cherng Shyu

**Affiliations:** 1 Department of Physical Therapy, Graduate Institute of Rehabilitation Science, China Medical UniversityTaichung, Taiwan; 2 Department of Healthcare Administration, Asia UniversityTaichung, Taiwan; 3 Graduate Institute of Clinical Medical Science, China Medical UniversityTaichung, Taiwan; 4 Translational Medicine Research Center, China Medical University HospitalTaichung, Taiwan; 5 Graduate Institute of Immunology, China Medical UniversityTaichung, Taiwan; 6 Center for Neuropsychiatry, Department of Neurology, China Medical University HospitalTaichung, Taiwan; 7 Department of Pathology, Buddhist Tzu-Chi General Hospital, Tzu-Chi UniversityHualien, Taiwan; 8 Department of Anesthesiology, China Medical University HospitalTaichung, Taiwan; 9 Institute of Molecular Biology, Academia SinicaTaipei, Taiwan; 10 Graduate Institute of Cancer Biology, Center for Molecular Medicine, China Medical UniversityTaichung, Taiwan; 11 Department of Biotechnology, Asia UniversityTaichung, Taiwan

**Keywords:** bone marrow derived cells (BMDCs), cell trafficking, hypoxia inducible factor 1α (HIF-1α), stress inducible protein type 1 (STI-1), stroke

## Abstract

Stress-inducible protein-1 (STI-1) is the proposed ligand for the cellular prion protein (PrP^C^), which is thought to facilitate recovery following stroke. Whether STI-1 expression is affected by stroke and how its signalling facilitates recovery remain elusive. Brain slices from patients that died of ischemic stroke were collected for STI-1 immunohistochemistry. These findings were compared to results from cell cultures, mice with or without the PrP^C^ knockout, and rats. Based on these findings, molecular and pharmacological interventions were administered to investigate the underlying mechanisms and to test the possibility for therapy in experimental stroke models. STI-1 was upregulated in the ischemic brains from humans and rodents. The increase in STI-1 expression *in vivo* was not cell-type specific, as it was found in neurons, glia and endothelial cells. Likewise, this increase in STI-1 expression can be mimicked by sublethal hypoxia in primary cortical cultures (PCCs) *in vitro*, and appear to have resulted from the direct binding of the hypoxia inducible factor-1α (HIF-1α) to the STI-1 promoter. Importantly, this STI-1 signalling promoted bone marrow derived cells (BMDCs) proliferation and migration *in vitro* and recruitment to the ischemic brain *in vivo*, and augmenting its signalling facilitated neurological recovery in part by recruiting BMDCs to the ischemic brain. Our results thus identified a novel mechanism by which ischemic insults can trigger a self-protective mechanism to facilitate recovery.

This work identifies HIF-1α-mediated transcription of STI-1 and PrPc interaction as leading to BMDCs recruitment into ischemic brains following stroke in both patients and animal models of stroke, highlighting novel neuroprotective possibilities.

## INTRODUCTION

Stress-inducible protein 1 (STI-1), also known as the heat shock protein-organizing protein (Hop) (Blatch et al, 1997), is a proposed ligand for the PrP^C^ (Martins et al, 1997; Zanata et al, 2002). Importantly, activation of PrP^C^ by STI-1 or an STI-1 mimetic peptide enhances neuronal protein synthesis (Roffe et al, 2010), promotes neurite outgrowth (Lopes et al, 2005) and protects neurons against apoptotic cell death (Lopes et al, 2005; Martins et al, 1997; Zanata et al, 2002). This raises the possibility that recombinant STI-1 and the STI-1 mimetic peptide may be of therapeutic importance to neurodegenerative diseases. Indeed, PrP^C^ is upregulated following cerebral ischemia (Weise et al, 2004), and deletion of the PrP^C^ gene exacerbates stroke outcome (Weise et al, 2006). Likewise, overexpression of PrP^C^ protects the ischemic brain against cerebral infarction and promotes neurological recovery (Shyu et al, 2005; Weise et al, 2008). Nevertheless, the role of STI-1 in stroke recovery has not been investigated, with key questions remaining to be answered: (1) Does ischemia affects the expression of STI-1 like it did with the expression of PrP^C^? And if so, how? (2) Does STI-1-to-PrP^C^ interaction confer neuroprotection and stroke recovery solely by inducing neuroprotection via the neuronal anti-apoptotic pathway, or does other non-neuronal mechanisms take place?

Trafficking of bone marrow derived cells (BMDCs) to a site of ischemic injury is thought to promote functional recovery (Hess et al, 2002; Shyu et al, 2004; Toth et al, 2008). Mobilization of these BMDCs requires degradation of the bone marrow chemokine stromal cell-derived factor I (SDF-1), which normally retains these cells by binding to their membrane receptor protein CXCR4 (Petit et al, 2002). The mobilized BMDCs then migrate across the bone marrow endothelium, a process that requires matrix metalloprotease (MMP) activity (De Becker et al, 2007; Heissig et al, 2002; Pruijt et al, 1999), to enter the circulation (De Becker et al, 2007; Petit et al, 2002; Pruijt et al, 1999). The circulating BMDCs are recruited to the site of injury, which is abundant in SDF-1, MMP-2 and MMP-9, where they cross the endothelium to accumulate and differentiate into mature tissues (Bastianutto et al, 2007; Kollet et al, 2003).

Given that hypoxia inducible factor-1α (HIF-1α) is the central transcriptional mediator of the cellular response of hypoxia and ischemia (Semenza, 2003; Taie et al, 2009), and that HIF-1α functions by binding to the hypoxia response element (HRE) of gene promoters to regulate gene expression governing survival under stress (Semenza, 2003; Taie et al, 2009), we investigated in this study whether STI-1 could be regulated by hypoxia/ischemia through binding of the HIF-1α to a putative HRE on the STI-1 promoter. Moreover, based on the role of PrP^C^ in stroke recovery (Weise et al, 2004; Weise et al, 2006) and the therapeutic efficacy of PrP^C^ overexpression in experimental model of focal ischemia (Shyu et al, 2005; Weise et al, 2008), we further studied whether STI-1 may promote stroke recovery via activation of PrP^C^, and more importantly, whether this neuroprotection may come about in part through STI-1 mediated recruitment of BMDCs.

## RESULTS

### STI-1 is upregulated in the ischemic brains from human patients and animals

**Figure 1:**
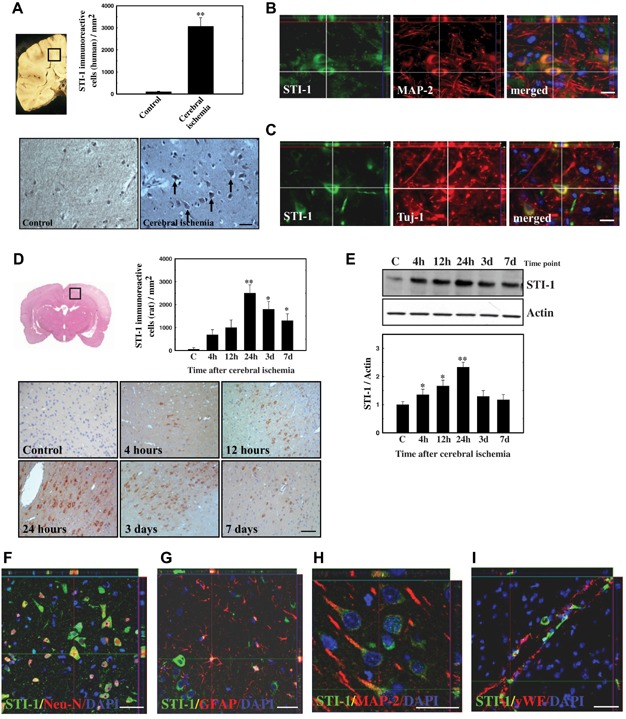
**Expression of STI-1 in the ischemic brains of humans and rats**Source data is available for this figure in the Supporting Information.Postmortem brain slices from human stroke patients (*n* = 4 patients) were stained for STI-1 immunoreactivity, and compared to control non-stroke patients died of glioblastoma multiforme (*n* = 4 patients). Tissue sampling was based on individual infarct topography, and infarction was identified macroscopically. About 1 cm^3^ of cortical sample was dissected for analysis. Significantly more STI-1^+^ cells were identified in the ischemic penumbra from stroke patients compared to similar area in control patients.Double-immunofluorescent microscopy showed that STI-1 was expressed in MAP-2^+^ neurons (B) and Tuj-1^+^ neurons (C) in the brain slices from stroke patients.The number of cells expressing STI-1 (D) and the total level of STI-1 expression (E) in the penumbral area of brains from rats subjected to cerebral ischemia were measured by immunohistochemistry and western blot, respectively. Cerebral ischemia increased the number of cells expressing STI-1 (D) and the total brain STI-1 expression (E) in a time-dependent manner, peaking at 24 h post-ischemia. *n* = 8 per rat group.Brain sections from rats subjected to cerebral ischemia were stained by immunohistochemistry, and analyzed with a Carl Zeiss LSM510 laser-scanning confocal microscope. STI-1 was co-expressed in Neu-N^+^ neurons (F), GFAP^+^ glia (G), MAP-2^+^ neurons (H) and vWF^+^ vascular endothelial cells (I) in the brains from rats 24 h post-cerebral ischemia. Values are mean ± SEM. (*p < 0.05 and **p < 0.01 versus control). Scale bar, 50 µm. See also Table 1.

To examine whether STI-1 play a role in the pathophysiology of cerebral ischemia, we collected postmortem brain tissues from human patients that died from fatal ischemic stroke 1–3 day post-ictus. For comparison, patients that died from non-ischemic causes served as control. Indeed, the penumbral region surrounding the ischemic infarct was populated by a high yield of STI-1-expressing cells (Table 1 and Fig 1A), which were extremely rare in the control brains. Moreover, double-immunofluorescent labelling revealed that these STI-1-expressing cells included MAP-2- and Tuj-1- expressing neurons (Fig 1B and C).

**Table:** Patient demography showing gender, age, cause of death and STI-1 immunoreactivity

		Gender	Age	Cause of death	STI-1 immunoreactivity (no. of STI-1^+^ cells)
Human stroke patients	1	M	54	Fatal cerebral infarction (3 days after onset)	3342 mm^−2^
	2	M	57	Fatal cerebral infarction (3 days after onset)	3617 mm^−2^
	3	F	59	Fatal cerebral infarction (1 day after onset)	2543 mm^−2^
	4	M	61	Fatal cerebral infarction (1 day after onset)	2762 mm^−2^
Control patients died of glioblastoma multiforme	1	M	60	Glioblastoma multiforme with brainstem failure	27 mm^−2^
	2	F	57	Glioblastoma multiforme with brainstem failure	31 mm^−2^
	3	F	61	Glioblastoma multiforme with brainstem failure	54 mm^−2^
	4	M	63	Glioblastoma multiforme with brainstem failure	49 mm^−2^

M, male; F, female; STI-1, stress-inducible protein 1.

To determine whether this increase in STI-1 expression was a specific result of the ischemic challenge, rats were subjected to cerebral ischemia, and at different time points post-ischemia, their brains were collected for immunohistochemical and western blot analysis. Consistent with findings from the human specimen, cerebral ischemia induced an abrupt increase in STI-1 expression near the penumbral region surrounding the infarct area (Fig 1D and E). Of interest, these STI-1^+^ cells are also especially prominent near the sub-ventricular regions and the peri-vascular regions of the ischemic brain (data not shown). In addition, the increase in STI-1 expression was time-dependent, and peaked at around 24 h post-ischemia (Fig 1D and E). Double-immunofluorescent labelling of brain slices from rats 24 h post-ischemia confirmed that these STI-1-expressing cells included Neu-N^+^ neurons (Fig 1F), GFAP^+^ glia (Fig 1G), MAP-2^+^ neurons (Fig 1H) and vWF^+^ vascular endothelial cells (Fig 1I).

### HIF-1α induced STI-1 expression by binding to the STI-1 promoter

**Figure 2:**
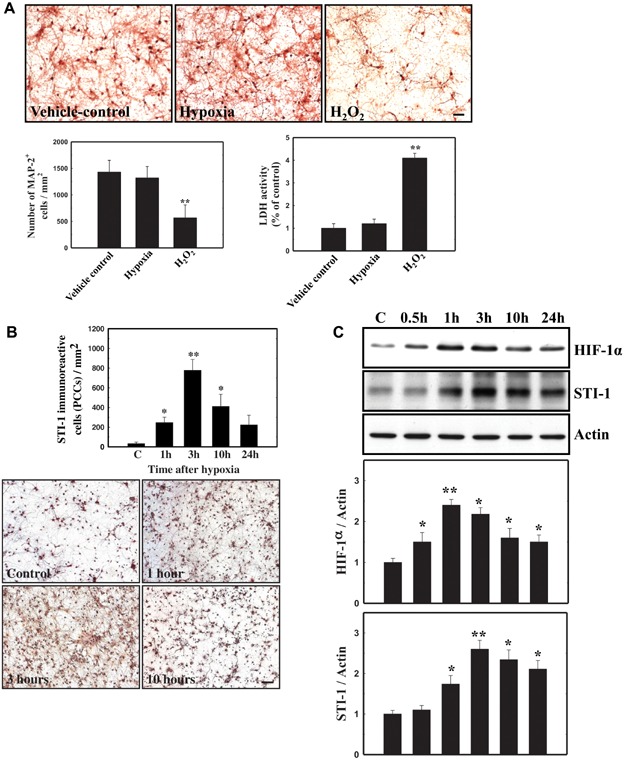
**Sublethal hypoxia induced STI-1 expression in primary cortical cultures (PCCs)**Source data is available for this figure in the Supporting Information.4 h-hypoxia in PCCs was sublethal. Hypoxia was induced in a two-gas incubator (Jouan, Winchester, Virginia) equipped with an O_2_ probe to regulate N_2_ levels. Represented images (*top panel*) and summarized data (*bottom left panel*) for the number of viable MAP-2^+^ neurons were shown. Summarized data based on lactate dehydrogenase (LDH) assay was also shown (*bottom right panel*).The number of PCCs expressing STI-1 (B) and the total level of STI-1 expression in the cultures (C) were measured by means of immunocytochemistry and western blot, respectively. Hypoxia increased the number of PCCs expressing STI-1 (B) and the total protein level of HIF-1α and STI-1 in the cultures (C). Values are mean ± SEM. (*p < 0.05 and **p < 0.01 versus control). Scale bar, 50 µm.

Given the pivotal role of HIF-1α in the cellular response to hypoxia and ischemia (Semenza, 2003), we asked the question whether non-lethal hypoxia can sufficiently upregulate STI-1 expression and whether such upregulation involves HIF-1α. Hypoxia was induced in primary cortical cultures (PCCs) by subjecting them to 4 h-incubation at 1% oxygen. We confirmed that this protocol was non-lethal by means of cell-counting and lactate dehydrogenase (LDH) assay, using hydrogen peroxide as a positive lethal control (Fig 2A). Consistent with our hypothesis, PCCs subjected to non-lethal 4 h-hypoxia also expressed HIF-1α and STI-1 in a time-dependent manner, which could be observed as early as 0.5 h post-hypoxia for HIF-1α and 1 h post-hypoxia for STI-1 (Fig 2B and C).

**Figure 3:**
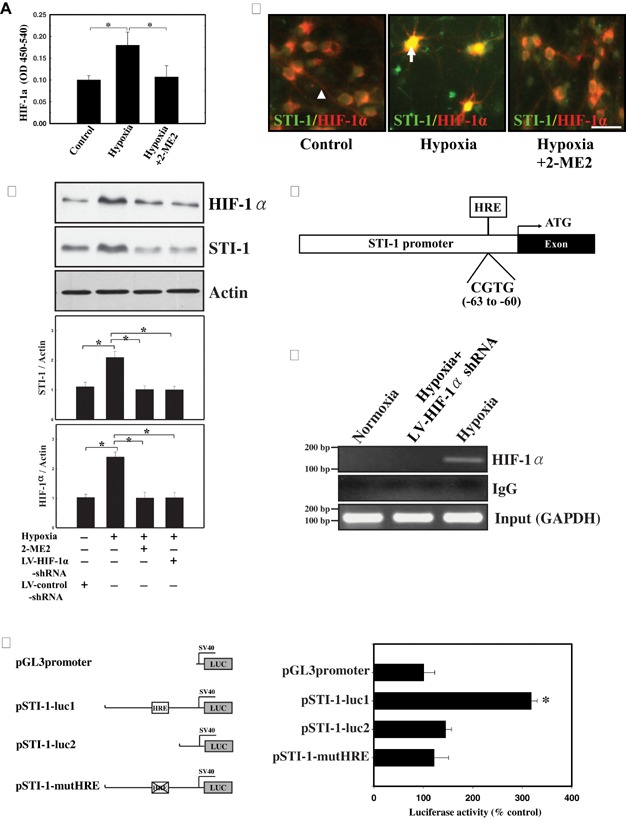
**Interaction between HIF-1α and the STI-1 promoter induced functional protein expression**Source data is available for this figure in the Supporting Information.HIF-1α activity, expressed in OD units (450–540 nm), was measured in nuclear extracts from primary cortical cultures (PCCs) by means of ELISA. Hypoxia (4 h-incubation in 1% O_2_) increased nuclear HIF-1α activity, and this was inhibited by the HIF-1α inhibitor 2-methoxyestradiol (2-ME2; 10 µM, 16 h pretreatment). *n* = 3 cultures per group.Immunofluorescent microscopy revealed the subcellular location of HIF-1α in PCCs subjected to 4 h-hypoxia with/without 2-ME2 (10 µM, 16 pretreatment) (arrow head indicates neurite, arrow indicates nucleus).Nuclear HIF-1α expression and whole-cell STI-1 expression in PCCs subjected to 4 h-hypoxia, HIF-1α inhibition by 2-ME2 (10 µM, 16 h pretreatment), and/or HIF-1α knockdown by lentiviral delivery of shRNA (LV-HIF-1α-shRNA) compared to LV-control-shRNA was measured by western blot.The schematic illustration of the STI-1 gene promoter showed a hypoxia response element (HRE) sequence: CGTG in the −63 to −60 position, for binding to the HIF-1α.Binding of HIF-1α to the STI-1 promoter was detected in PCCs subjected to hypoxia by chromatin immunoprecipitation (ChIP) assay. As a negative control, HIF-1α-knockdown by lentiviral delivery of the shRNA (LV-HIF-1α-shRNA) prevented detection of HIF-1α-to-DNA binding using ChIP.Functional protein expression by HIF-1α-to-DNA binding was detected following 4 h-hypoxia in 3T3 NIH cells expressing luciferase reporter gene constructs that either (1) contained the native STI-1 promoter (pSTI-1-luc1), (2) contained an HRE-lacking STI-1 promoter (pSTI-1-luc2), (3) contained an HRE-mutant STI-1 promoter (pSTI-1-mutHRE) or (4) lacking an STI-1 promoter (pGL3promoter). Values are mean ± SEM. (*p < 0.05 and **p < 0.01 versus control). Scale bar, 50 µm.

To test whether there is a causative relationship between HIF-1α and STI-1, we inhibited HIF-1α activation and nuclear translocation by the HIF-1α inhibitory reagent of 2-methoxyestradiol (2-ME2; 10 µM, pretreated for 16 h), which was found to downregulate the HIF-1α at the post-transcriptional level (Mabjeesh et al, 2003) (Fig 3A and B), and found that such HIF-1α inhibition prevented hypoxia-mediated STI-1 production (Fig 3B and C). Given that 2-ME2 is non-specific for HIF-1α inhibition, we repeated the experiment using knockdown technique. Here, HIF-1α knockdown by lentiviral delivery of the shRNA (LV-HIF-1α-shRNA) also prevented hypoxia-mediated STI-1 production compared to the LV-control-shRNA (Fig 3C). These data altogether suggested that HIF-1α was required for hypoxia-mediated STI-1 production.

Because HIF-1α is a transcription factor that works by binding to the HRE of gene promoters (Semenza, 2003), and the promoter region of the STI-1 gene contains a putative HRE (Fig 3D), we reasoned that HIF-1α upregulated STI-1 by binding to its promoter. Indeed, chromatin immunoprecipitation (ChIP) assay confirmed recruitment and direct binding of HIF-1α to the STI-1 promoter in PCCs subjected to 4 h-hypoxia, but not in PCCs subjected to 4 h-normoxia or PCCs treated with LV-HIF-1α-shRNA with 4 h-hypoxia (Fig 3E). To ensure that the binding of HIF-1α to the STI-1 promoter was functional, 3T3 NIH cells were transfected with a series of luciferase reporter gene constructs, either (i) containing the native STI-1 promoter (pSTI-1-luc1), (ii) containing an HRE-lacking STI-1 promoter (pSTI-1-luc2), (iii) containing an HRE-mutant STI-1 promoter (pSTI-1-mutHRE) or (iv) lacking an STI-1 promoter (pGL3promoter) (Fig 3F). In this luciferase reporter assay, 4 h-hypoxia significantly induced luciferase activity in cells transfected with pSTI-1-luc1, but not in cells transfected with either pGL3promoter, pSTI-1-luc2 or pSTI-1-mutHRE. This provides strong evidence that HIF-1α binding to the HRE of the STI-1 promoter functionally induced gene transcription and thereby protein expression.

### HIF-1α is required for STI-1 upregulation following stroke

**Figure 4:**
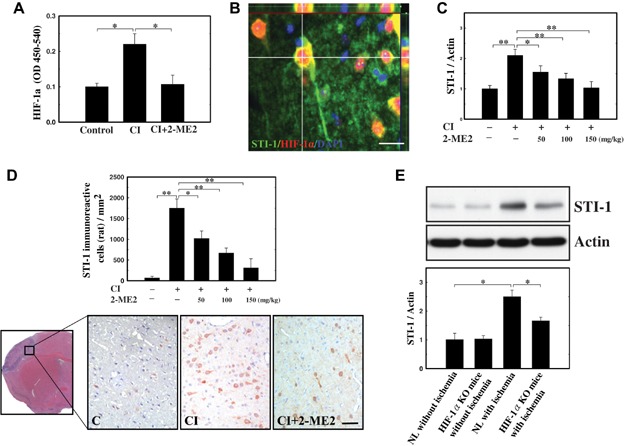
**Cerebral ischemia induced STI-1 expression in the rat brain via HIF-1α activation**Rats were treated with i.p. liposomal preparation of 2-ME2 (20 mg/mL) in different concentrations (50, 100 or 150 mg/kg) for 10 consecutive days before cerebral ischemia, and HIF-1α activity, expressed in OD units (450–540 nm), was measured in nuclear extracts from rat brains 4 h following cerebral ischemia. Cerebral ischemia (CI) increased nuclear HIF-1α activity, and this was inhibited by the HIF-1α inhibitor 2-methoxyestradiol (2-ME2; 100 mg/kg, i.p. daily for 20 days). *n* = 8 per rat group.Double-immunofluorescent microscopy of brain slices from rats 24 h post-cerebral ischemia revealed STI-1 expression (green) in cells with HIF-1α nuclear translocation (red). Nuclei were labelled with DAPI (blue).STI-1 expression level (C) and the number of cells expressing STI-1 (D) in rats 24 h post-cerebral ischemia was dose-dependently inhibited by 2-ME2 (i.p. daily for 20 days). *n* = 8 per rat group.STI-1 expression level in normal littermate (NL) and HIF-1α-knockout (HIF-1α-KO) mice 24 h post-cerebral ischemia. *n* = 8 per mice group. Values are mean ± SEM. (*p < 0.05 and **p < 0.01 versus control). Scale bar, 50 µm.

Our *in vitro* findings suggest that hypoxia may upregulate STI-1 via HIF-1α-to-HRE binding. To further test this notion, rats were subjected to cerebral ischemia, and their brains were collected to measure HIF-1α activity by ELISA (Fig 4A), and STI-1 expression by immunofluorescence (Fig 4B), Western blot (Fig 4C) and immunohistochemistry (Fig 4D). Here, cerebral ischemia substantially increased HIF-1α activity 4 h post-ischemia, and this can be inhibited by the HIF-1α inhibitor 2-ME2 (100 mg/kg, i.p. daily for 20 days) (Fig 4A). Consistent with the role of HIF-1α in STI-1 expression, double-immunofluorescence of the brain slices from rats 24 h post-cerebral ischemia showed that cells expressing HIF-1α are also expressing STI-1 (Fig 4B). Importantly, this STI-1 expression was dose-dependently inhibited by 2-ME2 (i.p. daily for 20 days) (Fig 4C and D). Likewise, cerebral ischemia in normal littermate (NL) mice, but not in HIF-1α-knockout (HIF-1α KO) mice, induced substantial STI-1 expression 24 h post-ischemia (Fig 4E). Together, these data supported an essential role of HIF-1α in mediating the increase in STI-1 expression in the ischemic brain.

### STI-1-to-PrP^C^ signalling promoted BMDC proliferation and trafficking

**Figure 5:**
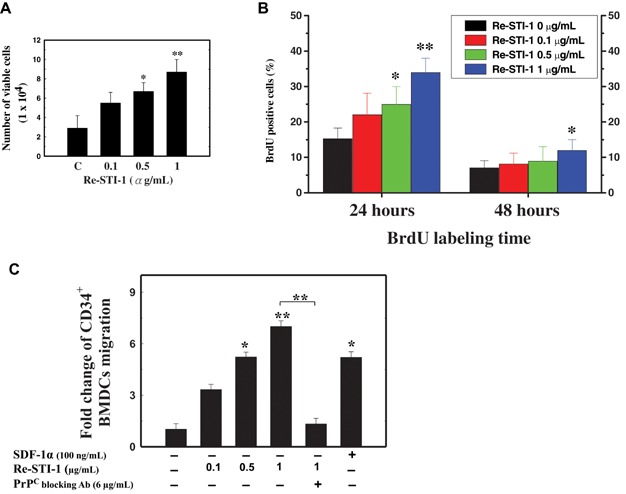
**STI-1 promoted bone marrow derived cell (BMDC) viability, proliferation and trafficking *in vitro***The effect of recombinant STI-1 (Re-STI1) was studied *in vitro* using CD34^+^ BMDC culture. Mobilized peripheral blood samples were collected from rat femoral veins (5 mL) mobilized with recombinant human granulocyte colony-stimulating factor (rHuG-CSF, Kirin), and CD34^+^ BMDCs were isolated by magnetic bead separation method. Re-STI-1 *in vitro* concentration-dependently (0.1–1 µg/mL) increased BMDC viability 12 h post-treatment, measured by trypan blue exclusion assay (A), and BMDC proliferation 24 h post-treatment, detected by bromodeoxyuridine (BrdU) labelling (B). *n* = 8 per group.Migration of CD34^+^ BMDCs treated STI-1 was assessed by transwell migration assays. Re-STI-1 *in vitro* concentration-dependently (0.1–1 µg/mL) increased CD34^+^ BMDC migration over a 4 h incubation period in a trans-well migration assay, and this was inhibited by the PrP^C^ blocking antibody (6H4, 6 µg/mL). Bone marrow chemokine stromal cell-derived factor 1α (SDF-1α, 100 ng/mL) served as a positive control. *n* = 8 per group.

**Figure 6:**
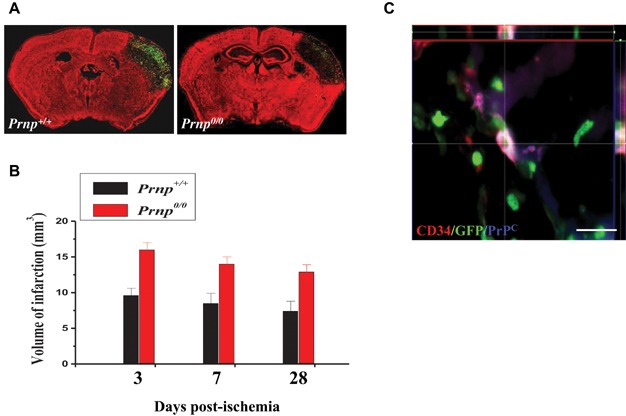
**PrP^C^ knockout impaired recruitment of bone marrow derived cell (BMDC) and exacerbated stroke damage *in vivo***Wild type (*Prnp*^*+/+*^) and PrP^C^-knockout (*Prnp*^*0/0*^) chimeric mice expressing GFP^+^ BMDCs were subjected to cerebral ischemia. GFP^+^ BMDCs were found in great abundance in the brains from *Prnp*^*+/+*^ mice, but not in brains from *Prnp*^*0/0*^ mice, 3 day post-cerebral ischemia.Volume of cerebral infarction in wild type (*Prnp*^*+/+*^) and PrP^C^-knockout (*Prnp*^*0/0*^) chimeric mice, measured by MRI at 3 day, 7 day and 28 day post-cerebral ischemia.3D immunofluorescent images showing co-expression of PrP^C^ in CD34^+^GFP^+^ BMDCs in the penumbric area of the brain. Scale bar, 50 µm.

In investigating whether STI-1 may promote stroke recovery in part through BMDC proliferation and trafficking, BMDC culture was prepared from mobilized peripheral blood samples from the rat femoral veins. In this BMDC culture, recombinant STI-1 (Re-STI-1; 0.1–1 µg/mL) concentration-dependently increased the number of viable cells 12 h post-treatment (Fig 5A), and this was in part due to an increase in the proliferation of these cells, as evident with a BrdU assay (Fig 5B). Moreover, in an *in vitro* transwell migration assay, Re-STI-1 (0.1–1 µg/mL) concentration-dependently recruited CD34^+^ BMDC, a sub-population of BMDCs, migration from the upper chamber to the lower chamber over a 4 h-period, and this Re-STI-1-mediated migration was blocked by a neutralizing antibody against PrP^C^ (6H4, 6 µg/mL) (Fig 5C). Based on the crucial role of BMDC migration in stroke recovery, we studied cerebral ischemia using wild type (*Prnp*^*+/+*^) and PrP^C^-knockout (*Prnp*^*0/0*^) GFP-chimeric mice containing GFP^+^ BMDCs (Fig 6A–C). In these animals, focal ischemia-mediated recruitment of the GFP^+^ BMDCs can be visualized in the brain slices under the fluorescent microscope. By 3 days after cerebral ischemia, it became clear that while ischemia induced substantial recruitment of these GFP^+^ BMDCs to the ischemic brain of *Prnp*^*+/+*^ mice, few GFP^+^ BMDCs were found in the ischemic brain of *Prnp*^*0/0*^ mice (Fig 6A). In addition, cortical infarct volume was larger in *Prnp*^*0/0*^ mice than that of *Prnp*^*+/+*^ mice, and in either species, cortical infarct volume progressively decreased in size from 3 to 28 day post-ischemia (Fig 6B). Consistent with the role of PrP^C^ in BMDC activity, some CD34^+^ GFP^+^ BMDCs in the penumbric area were found to co-express PrP^C^ (Fig 6C).

**Figure 7:**
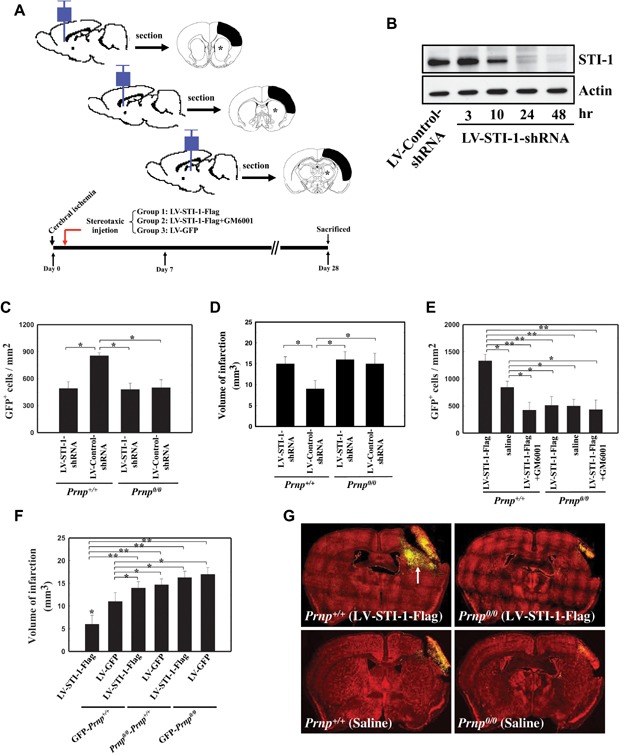
**STI-1 recruited bone marrow derived cell (BMDC) *in vivo***Source data is available for this figure in the Supporting Information.Schematic illustration of the experimental design. Mice were injected with lentivirus carrying vectors for STI-1 knockdown (LV-STI-1-shRNA) or overexpression (LV-STI-1) with or without the Flag-tag at regions of the brain shown.Western blot showing time course of STI-1 knockdown at different time points following lentiviral delivery of shRNA by intracerebral injection of LV-STI-1-shRNA (intracerebral bolus 30 min post-ischemia; 5 µL containing 10^10^ units).STI-1 knockdown by LV-STI-1-shRNA reduced the number of GFP^+^ BMDCs recruited to the ischemic brain in *Prnp*^*+/+*^ mice, measured by fluorescence microscopy 28 day post-cerebral ischemia (C) and increased cerebral infarct, detected by TTC-staining 3 day post-cerebral ischemia (D). In contrast, LV-STI-1-shRNA (intracerebral, 30 min post-ischemia) had no effect in *Prnp*^*0/0*^ mice, as these mice already had reduced GFP^+^ BMDCs, measured by fluorescence microscopy 28 day post-cerebral ischemia (C) and increased infarct, measured by TTC-staining 3 day post-cerebral ischemia (D). In addition, bar graph showed the cortical infarct volume in *Prnp*^*0/0*^ and *Prnp*^*+/+*^ mice from 3 to 28 days post-ischemia. *n* = 8 per mice group.STI-1-flag overexpression by LV-STI-1-Flag (5 µL containing 10^10^ units) increased the number of GFP^+^ BMDCs (arrow) recruited to the ischemic brain in *Prnp*^*+/+*^ mice, detected by fluorescence microscopy 28 day post-cerebral ischemia [(E), and representative images in (G)] and reduced cerebral infarct detected by TTC-staining 3 day post-cerebral ischemia (F). In contrast, LV-STI-1-Flag had no effect in *Prnp*^*0/0*^–*Prnp*^*+/+*^ chimeric mice and GFP-*Prnp*^*0/0*^ mice, as these mice continue to exhibit reduced GFP^+^ BMDCs, measured by fluorescence microscopy 28 day post-cerebral ischemia [(E), and representative images in (G)] and increased infarct detected by TTC-staining 3 day post-cerebral ischemia (F). Notably, recruitment of GFP^+^ BMDCs was also reduced by the MMP inhibitor GM6001 (100 mg/kg, i.p. daily × 5–8 day starting 4 h post-cerebral ischemia) (E). *n* = 8 per mice group. Values are mean ± SEM. (*p < 0.05 and **p < 0.01 versus control).

To investigate the role of STI-1 in stroke pathogenesis, we injected into the mice brain by lentivirus carrying vectors for STI-1 knockdown (LV-STI-1-shRNA) or overexpression (LV-STI-1) with or without the Flag-tag (Fig 7A). LV-STI-1-shRNA injected 30 min post-cerebral ischemia in *Prnp*^*+/+*^ mice decreased STI-1 protein expression in a time-dependent manner (Fig 7B), and in line with the role of STI-1 in BMDC activity and stroke recovery, LV-STI-1-shRNA decreased BMDC recruitment (Fig 7C) and exacerbated cerebral infarction 3 day post-ischemia (Fig 7D) to the level seen in *Prnp*^*0/0*^ mice. In marked contrast, LV-STI-1-Flag injected 30 min post-cerebral ischemia increased BMDC recruitment 28 day post-ischemia (Fig 7E and G) and reduced cerebral infarction 3 day post-ischemia (Fig 7F) in *Prnp*^*+/+*^ mice. Also, LV-STI-1-Flag failed to reduce infarct volume in the GFP-*Prnp*^*0/0*^ mice (chimeric *Prnp*^*0/0*^-mice expressing GFP/*Prnp*^*+/+*^-BMDCs) and the *Prnp*^*0/0*^–*Prnp*^*+/+*^ mice (chimeric *Prnp*^*+/+*^-mice with *Prnp*^*0/0*^-BMDCs) compared to that of control (Fig 7F), suggesting that neuroprotection by STI-1 required its dual-actions on both PrP^C^ from BMDC and PrP^C^ from non-BMDC host cells. Moreover, in line with the notion that MMPs are required for BMDC migration, the MMP inhibitor GM6001 (100 mg/kg, i.p. daily × 5–8 days, starting 4 h post-cerebral ischemia) prevented BMDC recruitment in these mice (Fig 7E).

### STI-1 facilitated stroke recovery by BMDC trafficking

**Figure 8:**
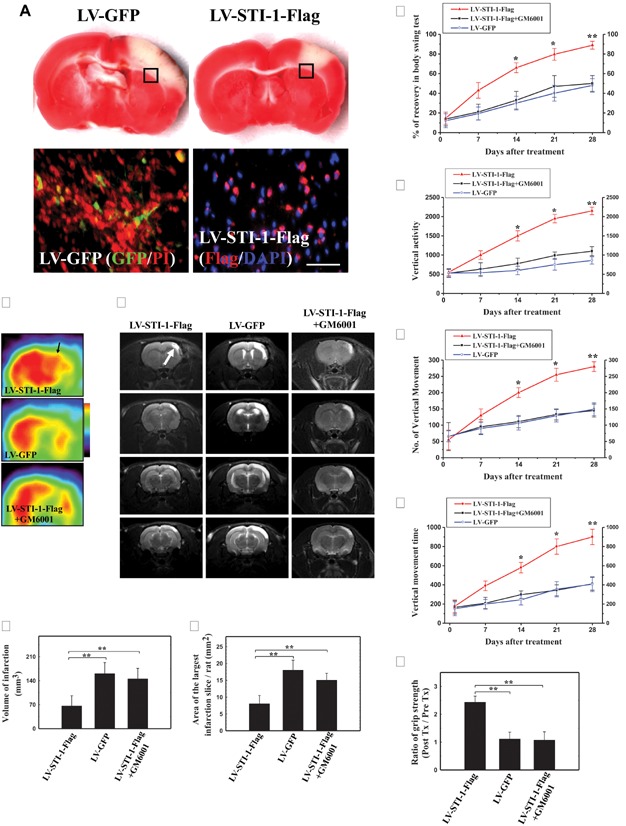
**STI-1 promoted functional recovery following cerebral ischemia in an MMP-dependent manner**LV-STI-1-Flag reduced infarct area outlined by TTC-staining 3 day post-cerebral ischemia (*top panels*). Widespread expression of GFP by LV-GFP (green, bottom left panel) and Flag by LV-STI-1-Flag (red, bottom right panel) was evident 7 day post-cerebral ischemia. *n* = 8 per rat group.Relative metabolic activity was determined using microPET scanning of [^18^F]fluoro-2-deoxyglucose (FDG) (Arrow indicated the right cortical area). LV-STI-1-Flag improved glucose metabolism, measured by FDG-PET in rats 7 day post-cerebral ischemia, and this was inhibited by MMP inhibitor GM6001 (100 mg/kg, i.p. daily × 5 day starting 4 h post-cerebral ischemia). *n* = 8 per rat group.Infarct volume following cerebral ischemia was determined using MRI in an imaging system (R4, GE) at 3.0 T. Infarct area in the right cortex was obtained by subtracting the non-infarct area in the right cortex from the total cortical area of the left hemisphere. The volume was then calculated by an internal volume analysis software. LV-STI-1-Flag decreased volume of cerebral infarction (D) and the largest infarct area (E), measured by MRI in rats 7 day post-cerebral ischemia, and this was inhibited by the GM6001. Representative images in (C). *n* = 8 per rat group.LV-STI-1-Flag improved body symmetry measured by swing test over 0–28 day post-cerebral ischemia (F), vertical activity over 0–28 day post-cerebral ischemia (G), number of vertical movements over 0–28 day post-cerebral ischemia (H), vertical movement time over 0–28 day post-cerebral ischemia (I), and grip strength 28 day post-cerebral ischemia (J), and these were inhibited by GM6001. *n* = 8 per rat group. Values are mean ± SEM. (*p < 0.05 and **p < 0.01 versus control). Scale bar, 50 µm.

**Figure 9:**
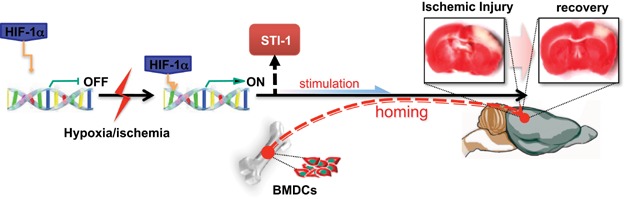
**HIF-1α-induced STI-1 enhanced BMDCs recruitment to facilitate neurological recovery** Schematic illustration showed that during cerebral ischemia, binding of HIF-1α to the STI-1 promoter triggered an upregulation of STI-1, which acted as a neuro-protective mechanism to reduce brain damage. Moreover, this could occur in part by promoting BMDC proliferation and/or trafficking.

Recruitment of BMDCs to the ischemic brain can play a crucial role in recovery (Asahara et al, 1997; Hess et al, 2002; Shyu et al, 2004; Toth et al, 2008), and our findings suggest that STI-1 may play a role in the recruitment of BMDCs. To further explore this mechanism, we overexpressed the rat brain with Flag-tagged STI-1 by intracerebral injection of LV-STI1-Flag 30 min post-cerebral ischemia (distribution shown in Fig 8A), and found substantial neuroprotection and behavioural recovery compared to control rats receiving LV-GFP instead (Fig 8A–J). This result was demonstrated by TTC staining at 3 day post-cerebral ischemia (Fig 8A), measuring glucose metabolism by FDG-PET scan at 7 day post-cerebral ischemia (Fig 8B), MRI infarct measure at 7 day post-cerebral ischemia (Fig 8C represented figures, and Fig 8D and E, summarized data), and neurological outcomes at 0–28 day post-cerebral ischemia (Fig 8F–J). More importantly, we found that STI-1 mediated neuroprotection was abolished by the MMP inhibitor GM6001 (100 mg/kg, i.p. daily for 5 day), suggesting that STI-1 mediated protection in part by promoting BMDC proliferation and/or trafficking. Taken together, our data suggests that during cerebral ischemia, binding of HIF-1α to the STI-1 promoter triggers an upregulation of STI-1, which act as a self-protective mechanism to reduce brain damage. Moreover, this could occur in part by promoting BMDC proliferation and/or trafficking (schematic illustration shown in Fig. 9).

## DISCUSSION

Although the biological importance of STI-1 is not completely understood, it appears that this protein may play a critical role in cellular response to environmental stress (Blatch et al, 1997; Nicolet & Craig, 1989). As such, STI-1-to-PrP^C^ signalling promotes a myriad of biological responses associated with cellular growth and survival (Lopes et al, 2005; Martins et al, 1997; Zanata et al, 2002). In line with these findings, we report novel evidence here that STI-1 is upregulated in the ischemic brains from human stroke patients, and that this can be mimicked by focal ischemic stroke in laboratory animals. Given that STI-1 is a proposed ligand for PrP^C^ (Martins et al, 1997; Zanata et al, 2002) and that PrP^C^ is also upregulated following stroke (Weise et al, 2004), the increase in STI-1 would undoubtedly complement the increase in PrP^C^, resulting in a substantial increase in STI-1-to-PrP^C^ signalling following stroke. Consistent with the findings that PrP^C^ deletion is deleterious (Weise et al, 2006) and that PrP^C^ overexpression is neuroprotective (Shyu et al, 2005; Weise et al, 2008) following stroke, our data provides evidence that STI-1 upregulation is crucial to stroke recovery. Importantly, we found that in addition to direct neuroprotection via the anti-apoptotic pathways, STI-1-to-PrP^C^ signalling may facilitate stroke recovery in part by recruiting BMDCs to the ischemic brain.

The mechanism by which ischemia upregulates STI-1 may be of particular interest. First, given that STI-1 was found to be upregulated in multiple distinct cell types, including neurons, glia and even endothelial cells, the mechanism by which ischemia upregulated STI-1 can probably be generalized to different tissues and organ parts, rather than being cell-type specific. Second, HIF-1α was the major drive behind STI-1 upregulation. This raises the intriguing possibility that STI-1 may be a downstream mediator for the myriad of hypoxic responses triggered by HIF-1α (Semenza, 2003; Taie et al, 2009), especially in relation to hypoxic preconditioning-induced cytoprotection against stroke (Giusti & Fiszer de Plazas, 2012; Taie et al, 2009; Valsecchi et al, 2011), heart failure (Cai et al, 2008; Eckle et al, 2008; Loor & Schumacker, 2008; Rane et al, 2009), liver failure (Knudsen et al, 2011), kidney failure (Weidemann et al, 2008; Zhang et al, 2011) and so forth. Third, STI-1 promoted BMDC viability and proliferation, and also promoted the recruitment of BMDCs into the ischemic brain. This suggests that STI-1 may mediate many of the known effects of HIF-1α on cell survival and proliferation (Hu et al, 2008; Kim et al, 2009; Liu et al, 2010; Takamiya et al, 2006; Wang et al, 2008). Future studies should look into how HIF-1α-to-STI-1 signalling participates in other physiological and pathological responses mediated by HIF-1α, and in different tissue types and organ parts.

In addition to direct neuroprotection by upregulating anti-apoptotic proteins, we found that STI-1/PrP^C^ signalling may promote stroke recovery by recruiting BMDCs into the ischemic brain. In our *in vitro* BMDC culture, STI-1 promoted BMDC survival, proliferation and trans-well migration. Consistent with the requirement of MMP activity in the migration of BMDCs across the endothelium (De Becker et al, 2007; Heissig et al, 2002; Pruijt et al, 1999) and to enter the site of injury where they differentiate into mature tissues (Bastianutto et al, 2007; Kollet et al, 2003), we report here that MMP activity is required for STI-1-mediated recruitment of BMDCs into the ischemic brain *in vivo*. Importantly, inhibition of STI-1-mediated BMDC recruitment with the specific MMP inhibitor GM6001 prevented recovery of the ischemic brain and behavioural outcome in an animal model of stroke. Thus, we reported the first evidence that STI-1 may increase the recruitment of BMDCs into the ischemic brain in an MMPs 2/9-dependent manner, and that this is crucial to STI-1-mediated stroke recovery.

## MATERIALS AND METHODS

### Autopsy samples of human brain

We investigated autopsy brain specimens from four cases of fatal ischemic stroke (disease duration of 1–3 day) treated at the Department of Neurology, Buddhist Tzu-Chi General Hospital (Table 1). Autopsy was performed at a mean of 6 h after death (ranging from 4 to 12 h). Four patients who died of non-ischemic causes served as controls. The study protocol was approved by the Institutional Review Board and informed consents were signed by patients' relatives. Tissue sampling was based on individual infarct topography, which was determined on the basis of cerebrovascular anatomy and most recent MRI scan. Brain samples with variable degrees of infarction were identified macroscopically and about 1 cm^3^ cortical samples, including subcortical white matter, were dissected and fixed with formalin prior to embedding in paraffin or frozen at −70°C until analysis, as previously described (Lindsberg et al, 1996).

### Animals and *in vivo* stroke model

Adult male Sprague-Dawley rats (weight 250–300 g) were used in this study. All animal studies and surgical procedures were approved to perform using sterile/aseptic techniques in accordance to the Institutional Guidelines of the China Medical University for the Care and Use of Experimental Animals. Rats were anesthetized with chloral hydrate (0.4 g/kg i.p.) and subjected to cerebral ischemia. Ligation of the right middle cerebral artery (MCA) and bilateral common carotid arteries (CCAs) were performed by methods described previously (Shyu et al, 2005). The CCAs were clamped with non-traumatic arterial clips. The right MCA was ligated with a l0-0 nylon suture. After 90 min of ischemia, the suture on the MCA and the arterial clips on the CCAs were removed to allow reperfusion. Core body temperature was monitored with a thermistor probe (Hewlett-Packard Model 21090A probe, Hewlett-Packard Company, Andover, MA), and maintained at 37°C with a heating pad during anaesthesia. After recovery from anaesthesia, rat body temperature was maintained at 37°C with a heat lamp.

### Conditional HIF-1α knockout mice (HIF-1α KO mice)

HIF-1α KO mice carrying a loxP-flanked allele of HIF-1α were kind gifts provided by Dr. Johnson (Ryan et al, 2000). HIF-1α disruption in the HIF-1α KO mice was induced by feeding doxycycline at a dose of 2 mg/mL in 5% w/v sucrose solution from embryonic day 15 to postnatal day 1. They were anesthetized with chloral hydrate (0.3 g/kg, i.p.) and subjected to right MCA ligation and ipsilateral CCA clamping for 120 min.

### Transgenic GFP-chimeric mice and PrP^C^ knockout mice

In order to verify the enhancement of BMDCs mobilization and homing into brain, a bone marrow niche sample was removed from the long bones of adult male donor mice as previously reported (Hess et al, 2002). Both ends of the femur and tibia were penetrated using a syringe with a 25-gauge needle, and the marrow was flushed out with sterile saline. Total marrow from each femur was diluted to 1 mL then strained through a 30-µm Spectramesh (Fisher Scientific). Before bone marrow transplantation, recipient wild type (C57BL/6 mice-*Prnp*^*+/+*^ mice) and *Prnp*^*0/0*^ mice [Zurich I, a generous gift provided by Dr. C. Weissmann (Flechsig et al, 2003)] underwent whole body gamma irradiation with ^137^Cs using a Gammacell 40 irradiator (MDS Nordion). A total dose of 9 Gy (900 rads) was administered to ablate the whole bone marrow. The mice received rescuing bone marrow transplantations within 24 h of irradiation. Donor bone marrow either from GFP mice or *Prnp*^*0/0*^ mice was injected into the recipient animal's tail as an 80 µL cell suspension containing 3 × 10^6^ cells to generate the GFP-*Prnp*^*+/+*^, GFP-*Prnp*^*0/0*^ and *Prnp*^*0/0*^–*Prnp*^*+/+*^ chimeric mice. Three weeks after transplantation, mice were anesthetized with chloral hydrate (0.3 g/kg, i.p.) and subjected to right MCA ligation and right CCA clamping for 120 min, as previously described (Shyu et al, 2005).

### Primary cortical cultures (PCC) preparation

PCCs were prepared from the cerebral cortex of gestation day 17 embryos from wild type (C57BL/6 mice-*Prnp*^*+/+*^ mice) and *Prnp*^*0/0*^ mice as previously described (Wetzel et al, 2008). PCCs were maintained under serum-free conditions in neurobasal medium (Invitrogen), supplemented with B-27 supplement (2%; Invitrogen), glutamine (0.5 mM; Sigma), glutamate (25 mM; Sigma), penicillin (100 U/mL) and streptomycin (100 mg/mL; Invitrogen). At the 4th day *in vitro*, half of the medium was removed and replaced with fresh medium without glutamate, as indicated by the manufacturer. The PCCs were maintained in a humidified incubator at 37°C with 5% CO_2_. At the 7th day *in vitro*, PCCs were used for experimentation.

### Hypoxia procedure

PCCs (1 × 10^5^ mL^−1^) cultured at 37°C in 5% CO_2_-humidified incubators were treated in normoxic (21% O_2_) or hypoxic conditions (1% O_2_) for different time point as previously described (Ivanovic et al, 2000). Hypoxic cultures were cultivated in a two-gas incubator (Jouan, Winchester, Virginia) equipped with an O_2_ probe to regulate N_2_ levels. Cell number and viability were evaluated using trypan blue exclusion assay.

### Measurement of LDH activity and immunocytochemistry of MAP-2

PCCs were prepared in 24-well plates. After 20 min, H_2_O_2_ (10^−4^ mol/L) was applied to the medium for 24 h and culture media were collected for LDH activity assays as previously described(Koh & Choi, 1987). In brief, LDH activity (U/10^−3^ L) was calculated from the slope of the decrease in optical density at 340 nm over a 3-min time period. One unit of LDH activity is defined as the amount of enzyme that catalyzes the consumption of 1 × 10^−3^ mol of NADH per minute. For MAP-2 immunostaining, primary cortical cell cultures were washed with PBS and fixed with 1% paraformaldehyde. Then, the fixed cells were treated for 20 min with blocking solution (10 g/L BSA, 0.03% Triton X-100). Cultures were incubated overnight at 4°C with a monoclonal antibody against MAP-2 (1:1000; mouse monoclonal, BM). The bound primary antibody was visualized by the labelled streptavidin-biotin (LSAB) method (DAKO LASB-2 Kit, Peroxidase, DAKO). The immunostaining procedure and the method for quantification of MAP-2^+^ cell density has been described previously (Wang et al, 2001).

### Preparation of CD34^+^ bone marrow derived cells (BMDCs) culture

Mobilized peripheral blood samples were collected from rat femoral veins (5 mL) mobilized with recombinant human granulocyte colony-stimulating factor (rHuG-CSF, Kirin) at 50 µg/kg/day subcutaneously for 5 consecutive days. The peripheral blood mononuclear cells (PBMCs) were separated by Ficoll-Paque (1:3 dilution, StemCell Technologies). CD34^+^ BMDCs were isolated from 2 × 10^6^ PBMCs by a magnetic bead separation method (MACS; Miltenyi Biotec) according to the manufacturer's instructions. Subsequently, CD34^+^ BMDCs (purity > 95%, 10^6^ cell/mL) were cultured for 72 h in medium (StemSpan^TM^ H3000 and Cytokine Cocktail, StemCell Technologies) at 37°C in a humidified atmosphere of 5% CO_2_/95% air and antibiotics, and prepared for experiment. For bromodeoxyuridine (Brdu, Sigma) labelling and immunocytochemistry, the cells were pulsed with 10 µM BrdU for 4 h and fixed with 4% paraformaldehyde for 20 min as previously described (Molina-Hernandez and Velasco, 2008). In brief, DNA was denatured by treatment with 2.5 N HCl for 20 min at room temperature followed by 0.1 M boric acid treatment to neutralize the cells. Incorporated BrdU was detected with a mouse monoclonal anti-BrdU antibody (1:50, BD Biosciences) that was incubated with the cells overnight. The percentage of BrdU-positive cells was determined by counting under a phase contrast microscope and at least 500 cells per sample were scored.

### Total protein extraction and western blotting

Experimental animals were decapitated at 4 h, 12 h, 3 day and 7 day after reperfusion with 90 min MCA ligation. Three rats without MCA ligation were used as normal controls. Samples of ischemic cerebral cortex were taken from the peripheral region of infarct brains (penumbral area). Western blot analysis of STI-1 was performed on these samples. Briefly, ischemic brain tissue was homogenized and lysed in a buffer containing 320 mM sucrose, 5 mM HEPES, 1 µg/mL leupeptin, and 1 µg/mL aprotinin. Lysates were centrifuged at 13,000 g for 15 min. The resulting pellet was resuspended in a sample buffer (62.5 mM Tris-HCl, 10% glycerol, 2% SDS, 0.1% bromophenol blue and 50 mM DTT) and subjected to SDS-polyacrylamide gel (4–12%) electrophoresis. Then, the gel was transferred to a Hybond-P nylon membrane. This was followed by incubation with appropriately diluted antibodies of STI-1 (1:2000, Santa Cruz), HIF-1α (1:200; Novus Biologicals), Bcl-2 (1:200; Santa Cruz), Bcl-xL (1:200; Transduction Laboratories), Bad (1:200; Santa Cruz), Bax (1:200; Transduction Laboratories) and β-Actin (dilution 1:2000, Santa Cruz). Membrane blocking, primary and secondary antibody incubations and chemiluminescence reactions were conducted for each antibody individually according to the manufacturer's protocol. The intensity of each band was measured using a Kodak Digital Science 1D Image Analysis System (Eastman Kodak). The ratio of band intensity of each protein in western blots in comparison with the internal control was calculated.

### Immunohistochemical analysis

Experimental rats were re-anesthetized with chloral hydrate (0.4 g/kg, i.p.), and were decapitated at 4 h, 12 h, 3 day and 7 day after cerebral ischemia. Three rats without MCA ligation were used as normal controls. Rat brains were fixed by transcardial perfusion with saline, followed by perfusion and immersion in 4% paraformaldehyde, and embedded in paraffin. A series of adjacent 6-µm-thick sections were cut from each tissue block in the coronal plane, stained with H&E and analyzed by light microscopy (E600, Nikon). Immunostaining was performed using the LSAB method (DAKO LASB-2 Kit, Peroxidase; DAKO). Briefly, tissue on a saline-coated slide was twice placed in a boiling citrate buffer (pH 6, ChemMate, DAKO) for 5 min in a microwave oven at 750 W after deparaffinization and rehydration, as previously described (8). This was followed by incubation with appropriate diluted antibodies to STI-1 (3) (1:1000, Santa Cruz) at room temperature for 1 h. After washing with Tris-buffered saline containing 0.1% Tween-20 (TBS-T), the specimens were sequentially incubated for 10–30 min with biotinylated anti-rabbit and anti-mouse immunoglobulins and peroxidase-labelled streptavidine. Staining was performed after 10 min incubation with a freshly prepared substrate-chromogen solution containing 3% 3-amino-9-ethylcarbazole and hydrogen peroxide. Finally, the slides were lightly counterstained with hematoxylin, washed with water, and then mounted. The extent of STI-1 cell immunoreactivity was measured as the number of cells per square millimeter (cells/mm^2^).

### Immunocytochemical analysis

Following hypoxia (1% O_2_ for 8 h), PCCs were collected for STI-1 immunostaining at each time point, and washed with PBS and fixed for 30 min at room temperature in 4% paraformaldehyde. After being washed with PBS, the fixed cultured cells were treated for 30 min with blocking solution (10 g/L BSA, 0.03% Triton X-100 and 4% serum in PBS). PCCs were incubated overnight at 4°C with a primary antibody against STI-1 (1:1000; mouse monoclonal, Santa Cruz), and secondary antibody conjugated with FITC fluorochromes (goat anti-mouse IgG) and then rinsed three times in PBS. FITC (green) fluorochromes on the immunofluorescence-labelled slides were excited by laser beam at 488 nm. The extent of STI-1 cell immunoreactivity was measured as the number of cells per square millimeter (cells/mm^2^). PCC expression of STI-1 was measured by western blot analyses using appropriately diluted antibodies to STI-1 as mentioned above.

### Laser-scanning confocal microscopy with double immunofluorescence

To identify the expression of cell type-specific markers in STI-1^+^ cells, double immunofluorescence was performed. Each coronal section was first stained with primary STI-1 antibody (1:1000; mouse monoclonal, Santa Cruz) and secondary antibody conjugated with FITC (1:500; goat anti-mouse IgG, Jackson Immunoresearch), followed by treatment with specific antibodies reacted to secondary antibody conjugated with Cy3 (1:500; goat anti-rabbit IgG, Jackson Immunoresearch), such as GFAP (1:400; rabbit polyclonal, Sigma), vWF (1:400; rabbit polyclonal, Sigma), Neu-N (1:200; rabbit polyclonal, Santa Cruz), microtubule-associated protein 2 (MAP-2, 1:200; rabbit polyclonal, Santa Cruz), Tuj-1 (1:200; rabbit polyclonal, Santa Cruz), hypoxia inducible factor-1α (HIF-1α, 1:200; rabbit polyclonal, Santa Cruz), CD34 (1:100, rabbit polyclonal, Santa Cruz) and Flag (1:400; rabbit polyclonal, Sigma). The tissue sections were analyzed with a Carl Zeiss LSM510 laser-scanning confocal microscope. FITC (green) and Cy3 (red) fluorochromes on the immunofluorescence labelled slides were excited by laser beam at 488 and 543 nm, respectively. STI-1, labelled with Cy3 (red) or FITC (green) fluorochromes, and cell-type-specific markers, Neu-N, MAP-2, Tuj-1, vWF, GFAP and HIF-1α, labelled with Cy3 (red) or FITC (green) fluorochromes were double immunostained in order to demonstrate their co-localization in one cell under laser-scanning confocal microscopy.

### Measurement of HIF-1α activity by ELISA

To measure the active HIF-1α, 50 µg nuclear extracts were incubated with biotinylated double stranded oligonucleotide containing a consensus HIF-1α binding site from Duo-set ELISA mouse active HIF-1α kit (R&D Systems) according to the manufacturer's instructions. The activity of HIF-1α was expressed by OD (450–540 nm) as previously described (Dai et al, 2007; Zanata et al, 2002). The experiments were carried out in triplicate unless otherwise mentioned.

### Chromatin immunoprecipitation (ChIP) Assay

To demonstrate the binding of HIF-1α protein to the promoter of human STI-1 (NCBI Accession number: NC_000011.9), the ChIP assay was performed with a commercial kit (Upstate Biotechnology) using the manufacturer's protocol with minor adjustments. The PCC were grown and incubated in air or 1% O_2_ for 8 h, and formaldehyde was added directly to culture medium followed by incubation for 10 min at 37°C as previously described (Ponnusamy et al, 2008). DNA–protein complexes were isolated on salmon sperm DNA linked to protein A agarose beads and eluted with 1% SDS, and 0.1 M NaHCO_3_. Cross-linking was reversed by incubation at 65°C for 5 h. Proteins were removed with proteinase K, and DNA was extracted with phenol/chloroform, re-dissolved and PCR-amplified (25 cycles) with STI-1 promoter primers, sense: 5′-GAACTCGACCAGTGAGCAGG-3′; and antisense: 5′-CCGTTGAATCGAATCCGTC-3′.

### Generation of promoter constructs, transient transfection and reporter gene assays

A fragment containing the 5′-flanking region (∼2200 bp) of the human *STI-1* gene promoter (NCBI Accession number: NC000011.9) was generated from human genomic DNA by PCR. This product was subcloned into the BamHI and SphI sites of the pGL3-basic vector (Promega) which contained one real HRE, and the generated plasmid was designated pSTI-1-luc1. One additional *STI-1* promoter construct (pSTI-1-luc2) without HRE was generated using the same downstream primer as for pSTI-1-luc1. In the pSTI-1-mutHRE construct, the putative HRE of pSTI-1-luc1 was replaced from 5′-CGTG-3′ to 5′-AAAG-3′ using the QuickChange Site-Directed Mutagenesis Kit (Stratagene). All constructs were verified by DNA sequencing. 3T3 NIH cells at about 90% confluence in 24-well plates were transiently transfected with reporter plasmid (0.5 µg) using Lipofectamine 2000 (Invitrogen) according to the manufacturer's instructions. To correct variable transfection efficiency, cells were co-transfected with the pRL-SV40 vector (0.05 µg) encoding the *Renilla* luciferase gene. Transfected cells were allowed to recover for 24 h in fresh medium, and then subjected to 1% O_2_ for 8 h. Cells were lysed and their luciferase activities were determined with a multiwell luminescence reader (Molecular Devices), by using the Dual-Luciferase Reporter Assay System (Promega).

### Transwell migration assays

Migration of CD34^+^ BMDCs (sub-population of BMDCs) treated STI-1 was assessed by transwell migration assays as previously described with modifications (Krankel et al, 2008). In brief, CD34^+^ BMDCs were placed in 100 µL in the upper chamber (transwell: 6.5-mm diameter, 5.0-mm pore size) according to manufacturer's instructions (Costar). Re-STI-1 (0.1, 0.5 and 1 µg/mL) or SDF-1α (positive control, 100 ng/mL, R&D System) was added in the lower chambers. For the PrP^C^ neutralizing antibody (6 µg/mL, 6H4, Prionics) studies, equal amount of antibodies were also added to the lower wells. The assays were conducted over a 4 h incubation period at 37°C in a 5% CO_2_ incubator. Because almost all cells stay at the lower side of the membrane after migration, quantification can be performed by simply counting these cells. Adhered cells at the lower side of the membranes were counted under the microscopy as previously described (Plett et al, 2002). For assessing migrated CD34^+^ cells, cells were collected from the bottom of a transwell and assessed by flow cytometry as previously described (Plett et al, 2002).

### Triphenyltetrazolium chloride (TTC) staining and immunofluorescent colocalized study

Three days after cerebral ischemia, animals were intracardially perfused with saline. The brain tissue was removed, immersed in cold saline for 5 min, and sliced into 2.0-mm-thick sections (seven slices per rat). The brain slices were incubated in 20 g/L TTC (Research Organics Inc), dissolved in saline for 30 min at 37°C, and then transferred into a 5% formaldehyde solution for fixation. The area of infarction in each slice was measured with a digital scanner, as previously described (Wang et al, 1997). The volume of infarction was obtained from the product of average slice thickness (2 mm) and by examining infarct areas in all brain slices. To minimize any artefacts induced by post-ischemic edema in the infarct tissue, the area of infarction was also calculated as previously described (Shyu et al, 2004). To measure the infarct area in the right cortex, we subtracted the non-infarct area in the right cortex from the total cortical area of the left hemisphere. Immunofluorescent colocalization analysis using the same method as described above with a specific antibody against Flag (1:200; Sigma) conjugated to FITC or Cy3 (1:300; Jackson Immunoresearch) was performed to demonstrate successful expression of STI-1 at either cell type in the ischemic brain following intracerebral injection of LV-STI-1-Flag. Further, the time course of STI-1-Flag expression after intracerebral injection of LV-STI-1-Flag in the ipsilateral ischemic brain was examined by Western blot using Flag (1:400; Sigma) antibody as described in the procedure above.

### Neurological behavioural measurements

Behavioural assessments were performed 3 days before cerebral ischemia, and 72 h after cerebral ischemia. The behavioural assessments contained (i) body asymmetry and (ii) locomotor activity (Shyu et al, 2004). Further, grip strength was analyzed using Grip Strength Meter (TSE-Systems) as previously described with modification (Dunnett et al, 1998). The baseline-test scores were recorded in order to normalize those taken after cerebral ischemia. The elevated body swing test was used to assess body asymmetry after cerebral ischemia and evaluated quantitatively as previously described (Shyu et al, 2004). Initially, animals were examined for lateral movement by suspending their bodies by their tails. The frequency of initial head swing contra-lateral to the ischemic side was counted in twenty continuous tests and was normalized, as follows: % recovery = [1-(lateral swings in 20 tests-10)/10 × 100%. Locomotor activity: rats were subjected to VersaMax Animal Activity monitoring (Accuscan Instruments) for about 2 h for behavioural recording. The VersaMax Animal Activity monitoring contained 16 horizontal and 8 vertical infrared sensors spaced 87 cm apart. The vertical sensors were situated 10 cm from the floor of the chamber. Motor activity was counted as the number of beams broken by a rat movement in the chamber. Three vertical parameters defined in the manufacturer's menu option were calculated over 2 h at night: (i) vertical activity, (ii) vertical time and (iii) number of vertical movements. In grip strength analysis, percentage of improvement in grip strength was measured on each forelimb separately and was calculated as the ratio between the mean strength out of 20 pulls of the side contralateral to the ischemia and the ipsilateral side. In addition, the ratio of grip strength post-treatment and baseline were also calculated and changes were presented as a percentage of baseline value.

### Measurement of infarct size using magnetic resonance image (MRI)

MRI was performed on rats under anaesthesia in an imaging system (R4, GE) at 3.0 T. Brains were scanned in 6–8 coronal image slices, each 2 mm thick without any gaps. T2-weighted imaging (T2WI) pulse sequences were obtained with the use of a spin-echo technique (repetition time, 4000 ms; echo time, 105 ms) and were captured sequentially for each animal at 3, 7 and 28 days after cerebral ischemia. To measure the infarct area in the right cortex, we subtracted the non-infarct area in the right cortex from the total cortical area of the left hemisphere. The area of infarct was drawn manually from slice to slice, and the volume was then calculated by an internal volume analysis software (Shyu et al, 2004) (Voxtool, General Electric).

### [^18^F]fluoro-2-deoxyglucose positron emission tomography (FDG-PET) examination

To assess the metabolic activity and synaptic density of brain tissue, experimental rats were examined using microPET scanning of [^18^F]fluoro-2-deoxyglucose (FDG) to measure relative metabolic activity under the protocol as previously described (Matsumura et al, 2003). In brief, ^18^F was produced by the ^18^O(p, n)^18^F nuclear reaction in a cyclotron at China Medical University and Hospital, Taiwan, and ^18^F-FDG was synthesized as previously described (Hamacher et al, 1986) with an automated ^18^F-FDG synthesis system (Nihon Kokan). Data were collected with a high-resolution small-animal PET (microPET, Rodent R4, Concorde Microsystems) scanner. The system parameters have been described previously by Carmichael (Carmichael et al, 2004). After one week of each treatment, animals were anesthetized with chloral hydrate (0.4 g/kg, i.p.), fixed in a customized stereotactic head holder and positioned in the microPET scanner. Then the animals were administrated with an intravenous bolus injection of ^18^F-FDG (200–250 µCi/rat) dissolved in 0.5 mL of saline. Data acquisition began at the same time and continued for 60 min using a 3-D acquisition protocol. The image data acquired from microPET were displayed and analyzed by Interactive Data Language (IDL) ver. 5.5 (Research Systems) and ASIPro ver. 3.2 (Concorde Microsystems) software. FDG-PET images were reconstructed using a posterior-based three-dimensional iterative algorithm (Kornblum et al, 2000) and overlaid on MR templates to confirm anatomical location (Brownell et al, 1998). Coronal sections for striatal and cortical measurements represented brain areas between 0 and +1 mm from bregma, and thalamic measurements represented brain areas between −2 and −3 mm from bregma, as estimated by visual inspection of the unlesioned side. The relative metabolic activity in regions of interest (ROI) of the striatum was expressed as a percentage deficit as previously described with modification (Carmichael et al, 2004).

### Preparation of lenti-viral constructs of STI-1

The lentiviral constructs were generated by co-transfection of human kidney derived 293T cells with three plasmids using the calcium phosphate method as previously described with modification (Salmon et al, 2000). In brief, the packaging construct contained the elongation factor-1α (EF-1α) promoter to express all the viral proteins. The second plasmid provided a vector with all the cis-acting elements to allow the transfer and integration of viral gene into the target cell's genome. In this transducing vector, an expression cassette with the Rev responsive element and the EF-1α promoter are used to direct the expression of STI-1-Flag (SC115872, OriGene) and GFP (LV-STI-1-Flag and LV-GFP). The third plasmid provides the envelope protein from the vesicular stomatitis virus glycoprotein to enhance viral stability and broaden the range of host cell types. The virus was harvested by collecting the cell culture medium after 48 h. After filtering the collected medium through 0.45-mm filters, the virus was concentrated by spinning at 4000 *g* for 15 min followed by a second spin (1000 *g*, 2 min at room temperature). The concentrated virus was stored at −80°C. The titer of lentiviral vectors was determined by dilution. The lentiviral titers were determined by infection of 293T cells seeded in six-well plates at 1 × 10^5^ cells per well the day before infection with serial dilution of the concentrated viral stock. After overnight incubation, the culture medium was changed and the cells incubated for two more days. GFP fluorescent cells were identified by fluorescent microscopy or by a fluorescent activated cell sorter. Titers ranged from 10^8^ to 10^9^ infectious units (IU)/mL.

### Stereotaxic injection of LV-STI-1-Flag and LV-GFP

The cerebral ischemic rat model was established as described above. Experimental rats were injected intracerebrally with 1 × 10^10^ virus units of LV-STI-1-Flag or LV-GFP (5 µL) 30 min after MCA ligation through a 26-gauge Hamilton syringe (Hamilton Company) into three cortical areas adjacent to the right MCA, 3.0–5.0 mm below the dura. The approximate coordinates for these injected sites were l.0–2.0 mm anterior to the bregma and 2.5–3.0 mm lateral to the midline, 0.5–l.5 mm posterior to the bregma and 3.5–4.0 mm lateral to the midline, and 3.0–4.0 mm posterior to the bregma and 4.5–5.0 mm lateral to the midline. The needle was retained in the same place for 5 min after each injection and a piece of bone wax was applied to the skull defects to prevent leakage of the injected solution. In addition, experimental mice were implanted stereotaxically with 1 × 10^9^ virus particles of LV-STI-1-Flag or LV-GFP through a 30-gauge Hamilton syringe into 2 cortical areas, 2.0–3.0 mm below the dura. The approximate coordinates for these sites were 0.5–1.0 mm anterior to the bregma and 1.5–2.0 mm lateral to the midline, and 1–l.5 mm posterior to the bregma and 2–2.5 mm lateral to the midline.

### Gene silencing of STI-1 and HIF-1α with RNA interference

*In vitro*, cells was infected with lentiviral particles of the LV-STI-1-shRNA (sc-153893-V, Santa Cruz), LV-HIF-1α-shRNA (sc-35562-V, Santa Cruz) or scramble LV-control-shRNA (sc-108080-V, Santa Cruz) under manufacture's instruction (Lukacs et al, 2010). For shRNA experiments *in vivo*, experimental animals were stereotaxically injected with LV-STI-1-shRNA or scramble LV-control-shRNA in accordance with the coordinates as above mentioned.

### 2-Methoxyestradiol treatment *in vivo* and *in vitro*

2-ME2 (Sigma) was dissolved in DMSO to obtain a 10 mmol/L stock solution. For *in vivo* experiments, the whole procedure was performed as previously described (Ricker et al, 2004). Experimental rats were treated with an intraperitoneal injection of a liposomal preparation (di-oleoyl-phosphotidylcholine; Avanti Polar Lipids) of 2-ME2 (20 mg/mL) in different concentrations (50, 100 or 150 mg/kg) for 10 consecutive days before and after cerebral ischemia. For *in vitro* experiments, PCC were pretreated with different concentrations of 2-ME2 (0.1, 1 and 10 µM) for 16 h as previously described (Dai et al, 2007).

### Blocking antibody of PrP^C^ and matrix metalloproteinases (MMPs) inhibitor

For the blocking experiment, experimental rats were treated with intraperitoneal injection of blocking antibody of PrP^C^ (6H4, 100 µg/rat/day, Prionics) or control IgG_2b_ (100 µg/rat) for 5–10 consecutive days (Lopes et al, 2005). In addition, 100 mg/kg of a broad, class-specific MMPs inhibitor (GM6001; Chemicon) was injected intraperitoneally for 5–8 consecutive days as previously described (Lee et al, 2006).

### Statistical analysis

All measurements in this study were performed blindly. Results are expressed as mean ± SEM. The behavioural scores have been evaluated for normality. We used two-way ANOVA with appropriate post hoc Newman–Keuls testing between different groups. A value of *p* < 0.05 was taken as significant.

Stroke has become one of the most severe medical issues of our day, with a burden to society that extends far beyond the personal tragedy. Understanding the molecular mechanisms mediating stroke-related brain damages and ways of how the brain damage can be repaired are urgently required to address this crisis. Recruitment (homing) of bone marrow derived cells (BMDCs) to ischemic brain areas following an ischemic brain insult has recently been suggested to play a critical role in improving neuronal survival and functional recoveries. However, the underlying mechanism remains poorly characterized.

RESULTS:

We report several novel discoveries that together reveal a previously unappreciated mechanism by which ischemia recruits BMDCs to the affected brain areas, thereby promoting morphological and functional recoveries. We first found that the expression of stress-inducible protein 1 (STI-1) was significantly up-regulated in the ischemic brain areas of stroke patients. Using a rat model of focal ischemia, we were able to demonstrate that the stroke-induced increased STI-1 expression is a result of activation of hypoxia-inducible factor-1α (HIF-1α)-mediated transcription. Importantly, we were able to demonstrate that this increase in STI-1 expression causally contributed to the increased homing of BMDCs to ischemic brain areas following stroke. Finally, using genetic mice lacking of prion protein (Prnp^0/0^) we characterized the underlying molecular cascades and found that STI-1 promotes the homing of BMDCs via its interaction with cellular prion protein (PrP^C^). Our results thus identified a novel mechanism by which ischemic insults can trigger a self-protective mechanism to facilitate neuronal survival, thereby reducing ischemic brain damage and enhancing functional recovery.

IMPACT:

This investigation identifies HIF-1α-mediated transcription of STI-1 and its interaction with PrPC as novel steps in the cascade leading to recruitment of BMDCs into ischemic brain areas following stroke in both human patients and in animal models of stroke, and thereby suggests that agents that enhance STI-1 expression and promote its interaction with PrPC may represent new class of neuroprotective therapeutics against stroke. In addition to acute brain insults such as stroke and brain trauma, chronic neuronal loss is believed to be a common cause for neuropathogenesis of many neurodegenerative diseases such as Parkinson's disease, Huntington's disease, amyotrophic lateral sclerosis and Alzheimer's disease. Thus, our study may lead to the development of STI-1 and PrPC interaction-based neuroprotective therapy for both acute brain injuries following stroke and neurotrauma and chronic brain damages associated with a large number of neurodegenerative diseases.

## Author contributions

WCS and YLY designed, conducted and supervised experiments and contributed to manuscript preparation; SDL conducted experiments and contributed to CD34^+^ BMDCs cultures, and lentiviral vector preparation; TWL contributed to experimental design, data analysis and interpretation, and took part in manuscript writing and preparation; CYL, SZL and CHL contributed to luciferase reporter cloning and animal studies; HJW and WL contributed to primary cortical cultures and immunohistochemical analysis; YHH, CYS contributed to human brain sample immunostating.

## Supporting Information

As a service to our authors and readers, this journal provides supporting information supplied by the authors. Such materials are peer reviewed and may be re-organized for online delivery, but are not copy-edited or typeset. Technical support issues arising from supporting information (other than missing files) should be addressed to the authors.
