# Supplementary material for: Role of stress-inducible protein-1 in recruitment of bone marrow derived cells into the ischemic brains
Source: EMBO Mol Med. 2013 Jul 8;5(8):1227–46. doi: 10.1002/emmm.201202258 (PMC3944463; doi:10.1002/emmm.201202258)
Supplement: Supplementary file 1 [file emmm0005-1227-SD1.pdf]

EMBO Molecular Medicine

Role of stress-inducible protein-1 in recruitment of bone marrow derived cells into the ischemic brains

Shin-Da Lee, Ted Weita Lai, Shinn-Zong Lin, Chen-Huan Lin, Yung-Hsiang Hsu, Chi-Yuan Li, Hsiao-Jung Wang, Wei Lee, Ching-Yuan Su, Yung-Luen Yu and Woei-Cherng Shyu

Corresponding author: Yung-Luen Yu and Woei-Cherng Shyu, China Medical University

Review timeline:

Submission date:	11 November 2012
Editorial Decision:	08 January 2013
Revision received:	11 April 2013
Editorial Decision:	02 May 2013
Revision received:	15 May 2013
Accepted:	17 May 2013

Transaction Report:

(Note: With the exception of the correction of typographical or spelling errors that could be a source of ambiguity, letters and reports are not edited. The original formatting of letters and referee reports may not be reflected in this compilation.)

Editor: Natscha Bushati / Céline Carret

1st Editorial Decision 08 January 2013

Thank you for the submission of your manuscript to EMBO Molecular Medicine and please accept my apologies for not replying earlier. We have now heard back from the three referees whom we asked to evaluate your manuscript.

You will see that while all three reviewers are generally supportive of your work and underline its potential interest, they also raise a number of specific concerns that would have to be convincingly addressed before further consideration.

- The mechanism whereby STI-1 recruits BMDCs is too unclear and would have to be better addressed. i.e. where is PrPC required, in the brain and/or BMDCs (referee #1)?

- Detailed analysis of the CD34+ cell population recruited by STI-1-PrP signalling would need to be undertaken (referees #1 and 2).

- Further data on infarct volume size at later time points would be required (referee #3).

- We would encourage you to substantiate the role of HIF-1a in regulation of STI-1 using primary cortical culture from HIF1-1a KO mice (referee #2).

Finally, all three reviewers raise a number of important suggestions to improve the interpretation of your data, the impact and readability of your work and to enhance its translational potential.

Given the potential interest of your study, we would be willing to consider a revised manuscript with

the understanding that the reviewers' concerns must be fully addressed, with additional experiments where appropriate.

Please note that it is EMBO Molecular Medicine policy to allow a single round of revision in order to avoid the delayed publication of research findings. Consequently, acceptance or rejection of the manuscript will depend on the completeness of your responses included in the next version of the manuscript.

EMBO Molecular Medicine has a "scooping protection" policy, whereby similar findings that are published by others during review or revision are not a criterion for rejection. I understand that the amount of work that would be required to submit a revised version of your manuscript is significant, hence, should you decide to submit a revised version, I do ask that you get in touch after three months if you have not completed it, to update us on the status.

Please also contact us as soon as possible if similar work is published elsewhere, as we then may not be able to extend the revision period beyond three months.

I look forward to receiving your revised manuscript in due course.

***** Reviewer's comments *****

Referee #1 (Comments on Novelty/Model System):

Please refer to my comments to the authors.

Referee #1 (General Remarks):

In this manuscript, Lee SD et al studied the role of stress-inducible protein-1 (STI-1), the ligand for the cellular prion protein (PrPc), in the ischemic stroke. Experimental evidence from rodent models, primary cortical and bone marrow (BM) cultures and histological analysis of human brain samples (from patients died of stroke) were provided. The authors suggest that STI-1 is upregulated in the ischemic brain tissue in multiple cell types, that it exerts a protective role by promoting the recruitment of BM derived cells (BMDCs) to the injury, and that overexpression of STI-1 in the ischemic brain can reduce infarct size and improve brain metabolism. While the overall concept is new and the mechanism underlying hypoxic induction of STI-1 expression is relatively strong, the evidence for the role of STI-1 in BMDC recruitment, i.e., the claimed reason for STI-1 to facilitate the recovery, is weak.

Specific comments:
1. The experiment of transplantation of GFP+ BMDCs to PrPc-knockout (Prnp0/0) recipients is rather confusion and does not establish the role of STI-1/PrPc axis in BMDCs, for which the authors need to show that transplantation of Prnp0/0 BMDCs to WT mice can impact the cell recruitment and ischemic stroke recovery.

2. Different subsets of BMDCs (e.g., progenitor cells vs. pro-inflammatory cells) can have opposite effects on ischemic neuronal recovery. However, it is not clear which populations of BMDCs are recruited to the ischemic brain by STI-1 upregulation. Also, the authors did not tell us what BMDCs express PrPc receptor and what are the levels.

3. BMDCs survival, proliferation, and migration are executed through different mechanisms; it is not conceivable that STI-1 promotes all these functions and does so solely through MMPs.

4. The blocking experiment of BMDC recruitment was performed by using the pan-MMP inhibitor GM6001. It is not clear to what extent the effects are attributable to BMDCs since MMPs are also critical for ischemic stroke recovery beyond BMDCs.

5. Since ischemia/hypoxia already can stimulate a dramatic increase in the level of STI-1 locally, it is not clear why administration of lentivirus-STI-1 AFTER cerebral ischemia can further increase the recovery in such a significant way (Figure 6).

6. Figure 1B-C: the authors need to provide figures with the overlay of the triple staining.

7. Figure 2C: A significant increase in STI-1 protein level occurred at just 1 h after hypoxia treatment (in primary cortical cultures). It is unlikely that this was due to the HIF-1 mediated transcriptional regulation. This needs clarification.

8. Figure 3B and 4B, immunofluorescent double staining of HIF-1 and STI-1 in the primary cortical cultures and in rat cerebral ischemic tissues, respectively: The hypoxic and ischemic cells showed an increase in HIF-1 nuclear translocation, however, this did not seem to be correlated with a greater level of STI-1 expression (i.e., the intensity of the STI-1 staining). Please use better representative pictures.

9. Figure 5E: this figure shows that in vivo administration of LV-STI-1 shRNA almost completely eliminated the expression of STI-1 by 48 h. What's the transduction efficiency of this virus?

Referee #2 (Comments on Novelty/Model System):

See my comments to the authors: some of the images are of low quality - should be improved.

Referee #2 (General Remarks):

Lee et al. demonstrate that stress inducible protein-1 (STI-1) is upregulated in a variety of cells in the ischeamic brain in humans as well as in rodents. The group has already shown that the overexpression of cellular prion protein (PrP) improves stroke recovery. In the present work they show that the upregulation of STI-1 is likely to be driven by the binding of the hypoxia inducible factor-1a (HIF-1a) to the STI-1 promoter. The induction of STI-1-PrP signaling results in recruitment of circulating bone marrow derived cells to the ischeamic region and improves stroke recovery. The work is very interesting and novel, but there are numerous problems that the authors need to attend to:
1. The authors isolate CD34+ cells from mobilized peropheral blood. They call these cells BMDC, but never make the distinction between hematopoietic (which is the cells they isolate) and stromal stem cells (MSCs or BMSCs) which have been studied and shown to have a beneficial effect in stroke recovery by many groups. Some of the studies that the authors refer to (for instance ref.14) actually are done using these cells and not the hematopoietic cells. It should be made clear which cells they use and the abbreviation should also be changed. BMDC would refer to any bone marrow derived cell - while the study exclusively uses hematopoietic and likely endothelial progenitors.
2. Why do the authors use 90 minutes of ischaemia in the rats and 120 min in the mice?
3. In the in vitro experiment the primary cortical cultures (PCC) are used to show the effect of hypoxia and the role of HIF-1a. The lentiviral shRNA knockdown experiment is not described in the Methods section. Was there a control used where scrambled shRNA was applied? Why did they not use a primary cortical culture from the HIF1-1a KO mice, that they used for the in vivo experiment?
4. Controls and details are not described for the immunostainings. Which species were the primary antibodies from? What kind of secondary antibodies were used? This information is very inportant to validate the double stainings that are performed.
5. The Figure legends should describe the depicted experiments in a way that one could understand what is shown, even without going back to read the relevant sections in the paper. This is not the case.
Fig.1 A: the scale is shown - but the legend does not mention what distance it represents (this is the case in several other Figures as well). The immunostaining shown in A is rather poor quality. Are the Control and the Cerebral ischaemia the same magnification?
What is the magnification in B and C? Once again, the brightfield ICC in D is too dark - although the staining is clear. A shorther exposure might help. The fluorescent stainings are nice.
Fig.2 A: the vehicle control image is out of focus and all three images are poor quality - the lighting is very uneven.
Fig.3 B: why is the STI-1 staining nuclear?
Fig.4 D: The stainings are very dark here, too.
Minor issue: there are several typos throughout the manuscript

Referee #3 (Comments on Novelty/Model System):

The current manusrcipt (S.-D. Lee et al.: Role of stress-inducible protein-1 in recruitment of bone marrow derived cells into the ischemic brains) addresses the influence of the hypothesized PrPc-ligand STI-1 in stroke. By the use of human infarcted brain tissue as well as in vivo and in vitro experimental stroke models, authors identified induction of STI1 expression. Furthermore, they investigated STI-1-related signalling mechanisms in experimental stroke and the effect on BMDC migration.
Based on the already described role of HIF1a sensing in stroke and the observed neuroprotective effects of the prion protein PrPc, authors examined the role of HIF in STI-1 expression regulation, as well as STI1/PrPc signaling in experimental stroke.
The topic is of high interest, and the paper is written straight-forward. However, some points of criticism remain:
Figure 3C: results of experiments using control shRNA should be added. Furthermore, declaration of 2ME2 as '..the HIF-1alpha inhibitor 2-methoxyestradiol..' (page 4/line 8) is misleading due to lack of specificity and should be omitted or the specificity of the inhibitor further discussed.
Figure 5I: infarct volumes (given in mm3) seem to be very small for the used model, authors should discuss, correct, or further specify this finding. Moreover, information about the infarct volume size at later time points (e.g. 28d as used for Figure 5H) would be very informative, as stroke volumes (and the degree of 'neuroprotection') may differ at different time points after induction of cerebral ischemia (e.g.: Bohacek et al. 2012 J Neuroinflamm 9:191).

Minor points:
. page 4 / line 10: instead of Fig3B: Fig.3C (?)
- Figure 4D: an overview showing the exact localization of the demonstrated regions within the hemisphere would be helpful.

1st Revision - authors' response 11 April 2013

Referee #1 (Comments on Novelty/Model System):

Please refer to my comments to the authors.

Referee #1 (General Remarks):

In this manuscript, Lee SD et al studied the role of stress-inducible protein-1 (STI-1), the ligand for the cellular prion protein (PrPc), in the ischemic stroke. Experimental evidence from rodent models, primary cortical and bone marrow (BM) cultures and histological analysis of human brain samples (from patients died of stroke) were provided. The authors suggest that STI-1 is upregulated in the ischemic brain tissue in multiple cell types, that it exerts a protective role by promoting the recruitment of BM derived cells (BMDCs) to the injury, and that overexpression of STI-1 in the ischemic brain can reduce infarct size and improve brain metabolism. While the overall concept is new and the mechanism underlying hypoxic induction of STI-1 expression is relatively strong, the evidence for the role of STI-1 in BMDC recruitment, i.e., the claimed reason for STI-1 to facilitate the recovery, is weak.

Specific comments:
1. The experiment of transplantation of GFP+ BMDCs to PrPc-knockout (Prnp0/0) recipients is rather confusion and does not establish the role of STI-1/PrPc axis in BMDCs, for which the authors need to show that transplantation of Prnp0/0 BMDCs to WT mice can impact the cell recruitment and ischemic stroke recovery.

Response: As requested by the reviewer, we have performed an experiment using newly generated chimeric $Prnp^{0/0}$-$Prnp^{+/+}$ mice (wild type $Prnp^{+/+}$ mice hosting $Prnp^{0/0}$ BMDCs). Like the GFP-$Prnp^{0/0}$ mice ($Prnp^{0/0}$ mice with GFP+ $Prnp^{+/+}$ BMDCs), these new mice also have exacerbated

stroke outcome that cannot be rescued by STI-1 overexpression (see Figure 7F). This suggests that PrP on both host tissue and BMDCs are important for STI-1-mediated protection and homing.

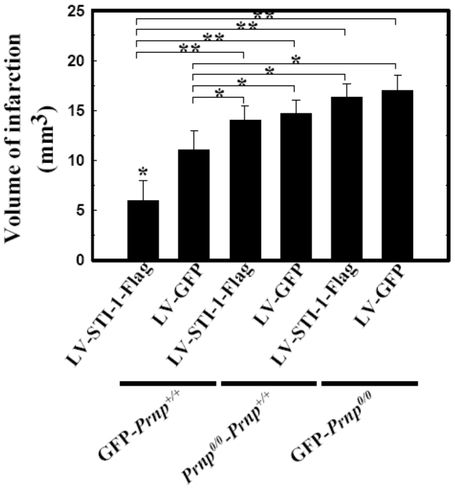

2. Different subsets of BMDCs (e.g., progenitor cells vs. pro-inflammatory cells) can have opposite effects on ischemic neuronal recovery. However, it is not clear which populations of BMDCs are recruited to the ischemic brain by STI-1 upregulation. Also, the authors did not tell us what BMDCs express PrPc receptor and what are the levels.

Response: $CD34^+$ hematopoietic stem cells (HSCs) are a significant sub-population of BMDCs in our experiment [and these notably co-express PrP^C (Blood 1998;91:1556-1561)], and our in vitro transwell migration experiment was carried out using $CD34^+$ BMDCs. Also, we perform a new immunohistochemical analysis on the rodent brain, which found about 10% $CD34^+GFP^+$ BMDCs co-expressed PrP^C in the pnumbric area (Figure 6C).

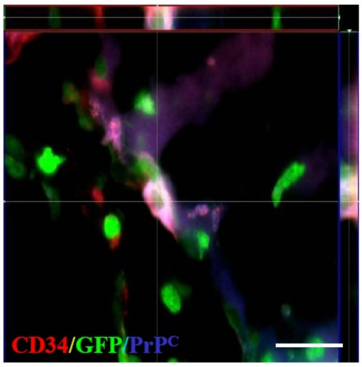

3. BMDCs survival, proliferation, and migration are executed through different mechanisms; it is not conceivable that STI-1 promotes all these functions and does so solely through MMPs.

Response: Like the results we are reporting here, other studies have demonstrated that STI-1 could promote cell survival, self-renewal, proliferation and migration in different type of cells (STEM CELLS 2011;29:1126–1136; PLoS ONE 2011;6:e18422; Cell Reports 2012;2:283–293; PNAS 2006;103:2184–2189; PLoS ONE 2012;7:e36389). Therefore, we find it conceivable that STI-1 really could enhance multiple functions. In addition, down-regulation of STI-1 by RNAi technique reduces the migration and invasion of pancreatic cancer cells, resulting from decreased expression of the downstream target gene, matrix metalloproteinases-2 (MMP2) (Cancer Letters 2011;306:180–189). Also, we only intended to show that STI-1 might enhance the BMDCs homing and migration through increasing the activity of MMP. But we did not show any data about the promotion of cell survival, self-renewal, and proliferation via STI-1-induced MMP.

4. The blocking experiment of BMDC recruitment was performed by using the pan-MMP inhibitor

GM6001. It is not clear to what extent the effects are attributable to BMDCs since MMPs are also critical for ischemic stroke recovery beyond BMDCs.

Response: Previous studies have shown that MMPs may mediate tissue injury by disrupting the blood-brain barrier (BBB) and causing neuronal death at 1-2 days after acute stroke stage. In the delayed stroke stage (3 days to week), MMPs may degrade inhibitory matrix substrates to release beneficial growth factors, as well as help in the recovery process (Nat Rev Neurosci. 2005;6:931–944; Stroke 2007;38:748-752; Journal of Neuroscience 2006;26:3491–3495). Migration and homing of BMDCs into ischemic brain might occur as early at 24 hours after stroke (Journal of Cerebral Blood Flow & Metabolism 2006:26:545–555; Leukemia 2011;25:1674–1686; Stem Cell Research & Therapy 2010;1:17). Therefore, MMPs plays the detrimental role during early stroke which is contrast to the function of recruited-BMDCs. In our study, since we use MMP inhibitor (GM6001, half life: one hour) starting at 4 hours post-ischemia, we assume that BMDC-induced effect might not affect by the MMPs in early stroke (occurring much later following stroke).

5. Since ischemia/hypoxia already can stimulate a dramatic increase in the level of STI-1 locally, it is not clear why administration of lentivirus-STI-1 AFTER cerebral ischemia can further increase the recovery in such a significant way (Figure 6).

Response: This situation is like that of Brain-derived neurotrophic factor (BDNF), which is present in large amounts in the adult brain where it plays a crucial role in plasticity and function (Neuroscience Letters 1996;211:57-60). Many studies consistently demonstrated that brain BDNF levels increased after stroke (Proc. Natl. Acad. Sci. U.S.A.1992;89:648–652; J. Cereb. Blood Flow Metab.1997;17:500–506], suggesting that BDNF promoted post-lesional plasticity and repair (J Cereb Blood Flow Metab 2005;25:281–290; Stroke 2009;40:1490–1495). Thus, brain-derived neurotrophic factor (BDNF) can decrease infarct volume and improve neurological outcome either by exogenously supplied or overexpression in *vivo* using genetic methods in experimental stroke (Stroke 2007;38:2165–2172; Stroke 2000;31:2212–2217; Int J Clin Exp Pathol 2011;4:496–504). In our previous studies (Circulation. 2004;110:1847-1854; J Clin Invest 2008;118:133–148; JPET 2008;324:834–849), we also provided the same evidence as above mentioned. Taken together, STI-1 could be another example, which was induced by the ischemic stress for applying in the treatment of stroke.

6. Figure 1B-C: the authors need to provide figures with the overlay of the triple staining.

Response: As reviewer's suggestion, we put the triple staining image in the Figures 1B and 1C.

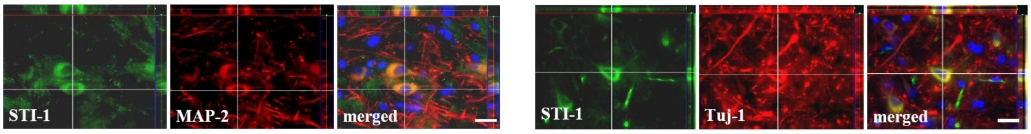

7. Figure 2C: A significant increase in STI-1 protein level occurred at just 1 h after hypoxia treatment (in primary cortical cultures). It is unlikely that this was due to the HIF-1 mediated transcriptional regulation. This needs clarification.

Response: As we knew, HIF-1α is an immediate early gene (JBC 2001;276:48754–48763). Thus, we do a new experiment to demonstrate the HIF-1α expression at 0.5 hour after early hypoxia (as previous article FEBS Letters 2000;468:53-58) and then STI-1 expression at 1 hour after hypoxia (Figure 2C).

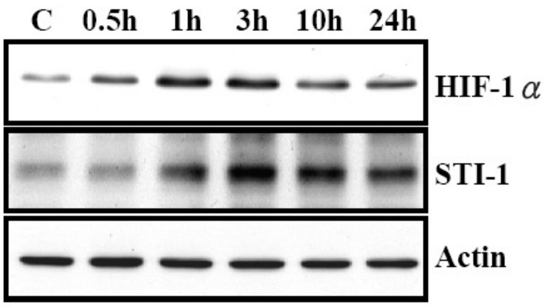

8. Figure 3B and 4B, immunofluorescent double staining of HIF-1 and STI-1 in the primary cortical cultures and in rat cerebral ischemic tissues, respectively: The hypoxic and ischemic cells showed an increase in HIF-1 nuclear translocation, however, this did not seem to be correlated with a greater level of STI-1 expression (i.e., the intensity of the STI-1 staining). Please use better representative pictures.

Response: As reviewer's suggestion, we improved the quality of the immunofluorescent staining in Figure 3B and 4B.

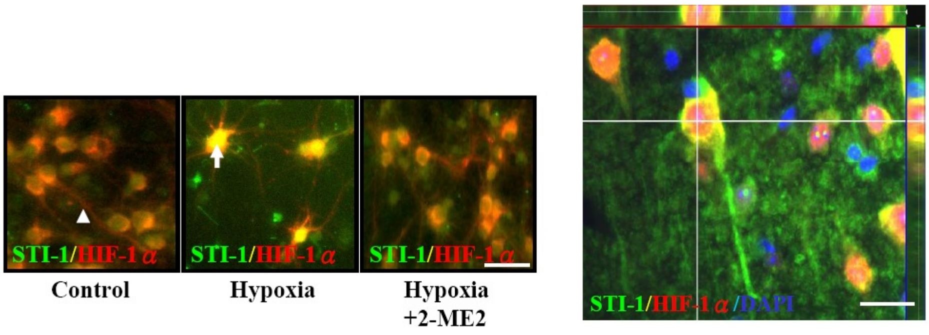

9. Figure 5E: this figure shows that in vivo administration of LV-STI-1 shRNA almost completely eliminated the expression of STI-1 by 48 h. What's the transduction efficiency of this virus?

Response: (Now Figure 7B) In our experience, lentivirus infections have very high transduction efficiency. Also, based on our LV-STI-1-Flag data and result of Figure 7B, transduction efficiency of LV-STI-1 shRNA for knockdown the STI-1 expression is close to 100% at the regions of injections.

Referee #2 (Comments on Novelty/Model System):

See my comments to the authors: some of the images are of low quality - should be improved.

Referee #2 (General Remarks):

Lee et al. demonstrate that stress inducible protein-1 (STI-1) is upregulated in a variety of cells in the ischaemic brain in humans as well as in rodents. The group has already shown that the overexpression of cellular prion protein (PrP) improves stroke recovery. In the present work they show that the upregulation of STI-1 is likely to be driven by the binding of the hypoxia inducible factor-1a (HIF-1a) to the STI-1 promoter. The induction of STI-1-PrP signalling results in recruitment of circulating bone marrow derived cells to the ischaemic region and improves stroke recovery. The work is very interesting and novel, but there are numerous problems that the authors need to attend to:

1. The authors isolate CD34+ cells from mobilized peripheral blood. They call these cells BMDC, but never make the distinction between hematopoietic (which is the cells they isolate) and stromal stem cells (MSCs or BMSCs) which have been studied and shown to have a beneficial effect in

© EMBO

stroke recovery by many groups. Some of the studies that the authors refer to (for instance ref.14) actually are done using these cells and not the hematopoietic cells. It should be made clear which cells they use and the abbreviation should also be changed. BMDC would refer to any bone marrow derived cell - while the study exclusively uses hematopoietic and likely endothelial progenitors.

Response: Yes, we agree with the reviewer's opinion. In our previous publications and others, we also found that circulating $CD34^+$ hematopoietic cells might be one of the major targets for cytokine or growth factors induced-mobilization (Circulation. 2004;110:1847-1854; J Clin Invest 2008;118:133–148; JPET 2008;324:834–849; JMCB 2012;4:184–187; Circulation. 2007;115:553-561). $CD34^+$ hematopoietic stem cells (HSCs) are a significant sub-population of BMDCs in our experiment, and our in vitro transwell migration experiment was carried out using $CD34^+$ BMDCs. While the $CD34^+$ cells contain major part of BMDCs (Prog Neurobiol. 2011;95:213–228), it is not the only BMDC cell population injected into the animal. Rather, the injected BMDC include predominantly $CD34^+$ but also other minor cell types. We use the term of "BMDCs" to avoid so strong tense, and to reflect the presence of other cell types.

2. Why do the authors use 90 minutes of ischaemia in the rats and 120 min in the mice?

Response: In rats' stroke model, we performed ligation of the right middle cerebral artery (MCA) and bilateral common carotid arteries (CCAs) for 90 mins, but in mice, we just performed ligation of the right MCA and right CCA for 120 mins. These protocols develop a uniform and reproducible stroke model (both in stroke volume and neurological disability) in our previous articles (Circulation. 2004;110:1847-1854; J Neurosci 2005;25:8967–8977; J Clin Invest 2008;118:133–148; J Clin Invest 2008;118:2482–2495; JMCB 2012;4:184–187).

3. In the in vitro experiment the primary cortical cultures (PCCs) are used to show the effect of hypoxia and the role of HIF-1a. The lentiviral shRNA knockdown experiment is not described in the Methods section. Was there a control used where scrambled shRNA was applied? Why did they not use a primary cortical culture from the HIF1-1a KO mice, that they used for the in vivo experiment?

Response: We added the detail method for the lentiviral shRNA knockdown experiment in the Methods section. Yes, we do use the scrambled shRNA for the control experiment. Since the conditional HIF-1α KO mice was induced by feeding doxycycline from embryonic day 15 to postnatal day 1, they are not appropriate for building PCCs at gestation day 17 embryos.

4. Controls and details are not described for the immunostainings. Which species were the primary antibodies from? What kind of secondary antibodies were used? This information is very important to validate the double stainings that are performed.

Response: We added the detail methods of immunostaining procedures in the Methods section. The primary antibodies were from mouse or rabbit species. The secondary antibodies were goat anti-mouse or goat anti-rabbit species (IgG). (page 21)

5. The Figure legends should describe the depicted experiments in a way that one could understand what is shown, even without going back to read the relevant sections in the paper. This is not the case.

Response: As reviewer's suggestion, we revised the description of each figure legend with more methods.

Fig.1 A: the scale is shown - but the legend does not mention what distance it represents (this is the case in several other Figures as well). The immunostaining shown in A is rather poor quality. Are the Control and the Cerebral ischaemia the same magnification? What is the magnification in B and C? Once again, the bright field ICC in D is too dark - although the staining is clear. A shorter exposure might help. The fluorescent stainings are nice.

Response: We did show the scale bar of 50 μm in each figure legend (last sentence). We also improve the quality of the Figure 1A, which revealed the same magnification between cerebral ischemia and control, as well as in Figure 1B and 1C.

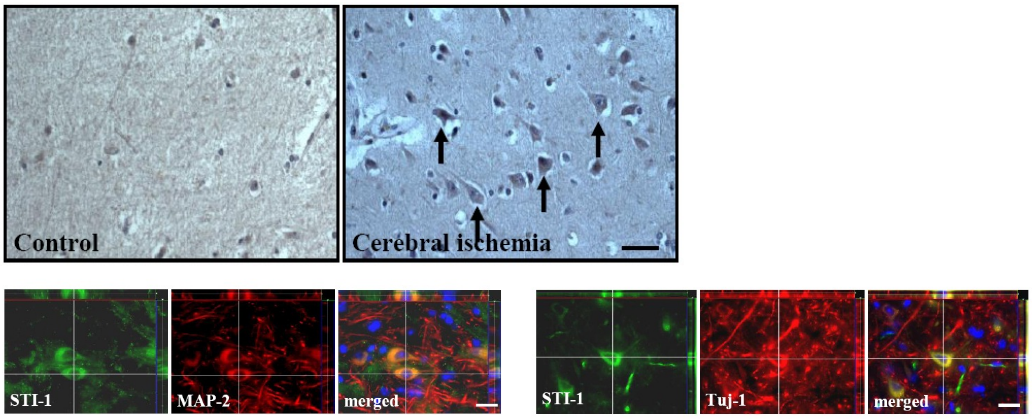

Fig.2 A: the vehicle control image is out of focus and all three images are poor quality - the lighting is very uneven.

Response: We improved the quality of the Figure 2A,

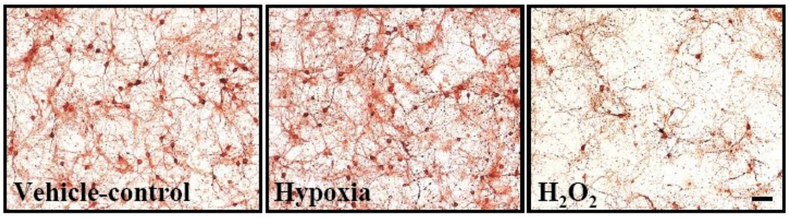

Fig.3 B: why is the STI-1 staining nuclear? Fig.4 D: The stainings are very dark here, too.

Response: Actually, STI-1 is expressed both at cytoplasm and nucleus of PCCs in our study. We also improved the quality of the staining in both Figure 3B and Figure 4D.

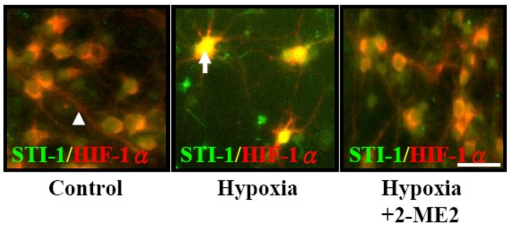

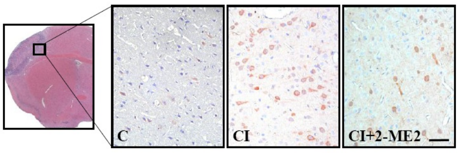

Minor issue: there are several typos throughout the manuscript

Response: We correct the typing errors in the text.

Referee #3 (Comments on Novelty/Model System):

The current manuscript (S.-D. Lee et al.: Role of stress-inducible protein-1 in recruitment of bone marrow derived cells into the ischemic brains) addresses the influence of the hypothesized PrPc-ligand STI-1 in stroke. By the use of human infarcted brain tissue as well as in vivo and in vitro experimental stroke models, authors identified induction of STI1 expression. Furthermore, they investigated STI-1-related signalling mechanisms in experimental stroke and the effect on BMDC migration.
Based on the already described role of HIF1a sensing in stroke and the observed neuroprotective effects of the prion protein PrPc, authors examined the role of HIF in STI-1 expression regulation, as well as STI1/PrPc signalling in experimental stroke.
The topic is of high interest, and the paper is written straight-forward. However, some points of criticism remain:

1.Figure 3C: results of experiments using control shRNA should be added. Furthermore, declaration of 2ME2 as '..the HIF-1alpha inhibitor 2-methoxyestradiol..' (page 4/line 8) is misleading due to lack of specificity and should be omitted or the specificity of the inhibitor further discussed.

Response: As reviewer's suggestion, we added LV-control-shRNA in the Figure 3C. We revised the "inhibitor" to "inhibitory reagent" to reduce the tense. As reviewer's suggestion, we declared that 2-ME2 is lack of specificity on HIF-1α inhibition in the Result section, and so we further used the shRNA knockdown method to demonstrate the consistent result (Page 8).

2.Figure 5I: infarct volumes (given in mm3) seem to be very small for the used model, authors should discuss, correct, or further specify this finding. Moreover, information about the infarct volume size at later time points (e.g. 28d as used for Figure 5H) would be very informative, as stroke volumes (and the degree of 'neuroprotection') may differ at different time points after induction of cerebral ischemia (e.g.: Bohacek et al. 2012 J Neuroinflamm 9:191).

Response: In mice stroke model, we performed ligation of the right MCA and right CCA for 120 mins, and then released the ligation suture for reperfusion. These protocols develop a uniform and reproducible stroke model, which show cortical infarction (both in stroke volume and neurological disability) in our previous articles (Circulation. 2004;110:1847-1854; J Neurosci 2005;25:8967–8977; J Clin Invest 2008;118:133–148; J Clin Invest 2008;118:2482–2495; JMCB 2012;4:184–187). Our model is different from that of Bohacek et al report which use middle cerebral artery occlusion (MCAO) method for one hour, whose infarct volume is larger than our model. As reviewer's suggestion, we provide bar graph to show the stroke volume of *Prnp*$^{+/+}$ and *Prnp*$^{0/0}$ mice at 3, 7 and 28 days post-ischemia at Figure 6B, which did not reveal the same phenomenon as Bohacek et al report.

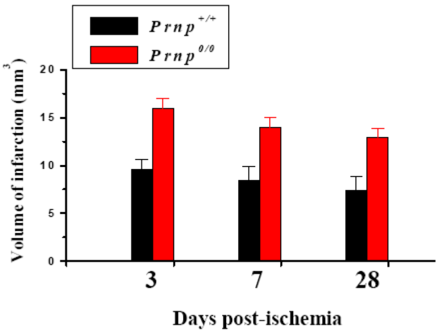

Minor points:
page 4 / line 10: instead of Fig3B: Fig.3C (?)

Response: We revised the incorrect label of Figure 3B to Figure 3B and 3C.

Figure 4D: an overview showing the exact localization of the demonstrated regions within the hemisphere would be helpful.

Response: As reviewer's suggestion, we revised to show the penumbric overview of the STI-1 immunostaining for the Figure 4D.

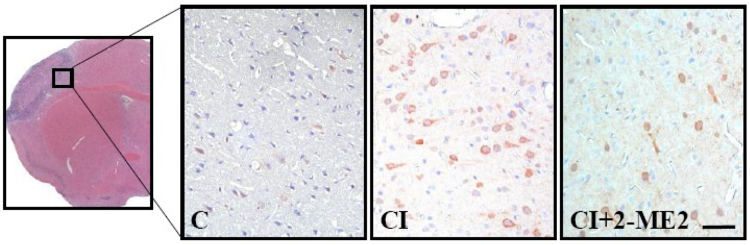

2nd Editorial Decision 02 May 2013

Thank you for the submission of your revised manuscript to EMBO Molecular Medicine. We have now received the enclosed reports from the referees that were asked to re-assess it. As you will see the reviewers are now globally supportive and I am pleased to inform you that we will be able to accept your manuscript pending the following final amendments:

-Please modify figure 1D as suggested by referee 2.

-Please amend the figure legends to increase impact and readability:
"Figure 1: Expression of STI-1 in the ischemic brains of humans and rats"
Use present or past tense in all legends to harmonize across all.

-We noted that you added figures in your point-by-point response to the referees. According to the EMBO Publications transparent editorial process initiative, we will publish a Review Process File online to accompany accepted manuscripts (please see below). Please let us know whether you are happy for us to include the figures or not.

-We now encourage the publication of source data, particularly for electrophoretic gels and blots, with the aim of making primary data more accessible and transparent to the reader. Would you be willing to provide a PDF file per figure that contains the original, uncropped and unprocessed scans of all or key gels used in the figure? The PDF files should be labeled with the appropriate figure/panel number, and should have molecular weight markers; further annotation may be useful but is not essential. The PDF files will be published online with the article as supplementary "Source Data" files. If you have any questions regarding this just contact me.

Please submit your revised manuscript within two weeks.

I look forward to reading a new revised version of your manuscript as soon as possible.

***** Reviewer's comments *****

Referee #1 (Comments on Novelty/Model System):

The quality of data has been improved in the revised manuscript.

Referee #1 (General Remarks):

No further questions.

Referee #2 (General Remarks):

The authors responded to the critiques. The work is significantly improved. There is one remaining problem that the authors can and should fix. In Fig.1, theD panel has 6 small pieces demonstrating brightfield ICC. These are way too dark. Maybe white-balancing them would do the trick. This portion is an eyesore in an otherwise high quality Figure.

Referee #3 (General Remarks):

The reviewer's concerns have been addressed sufficiently.

2nd Revision - authors' response 15 May 2013

Please modify figure 1D as suggested by referee 2.

Response: As requested, we have made the image brighter with white-balance.

-Please amend the figure legends to increase impact and readability: "Figure 1: Expression of STI-1 in the ischemic brains of humans and rats." Use present or past tense in all legends to harmonize across all.

Response: As requested, we revised the figure 1 legend title to "Expression of STI-1 in the ischemic brains of humans and rats", and unified the tense.

-We noted that you added figures in your point-by-point response to the referees. According to the EMBO Publications transparent editorial process initiative, we will publish a Review Process File online to accompany accepted manuscripts (please see below). Please let us know whether you are happy for us to include the figures or not.

Response: We are fine with the figure inclusion.

-We now encourage the publication of source data, particularly for electrophoretic gels and blots, with the aim of making primary data more accessible and transparent to the reader. Would you be willing to provide a PDF file per figure that contains the original, uncropped and unprocessed scans of all or key gels used in the figure? The PDF files should be labelled with the appropriate figure/panel number, and should have molecular weight markers; further annotation may be useful but is not essential. The PDF files will be published online with the article as supplementary "Source Data" files. If you have any questions regarding this just contact me.

Response: The source data is now included.
