# Supplementary figures and images for: Role of stress-inducible protein-1 in recruitment of bone marrow derived cells into the ischemic brains
Source: EMBO Mol Med. 2013 Jul 8;5(8):1227–46. doi: 10.1002/emmm.201202258 (PMC3944463; doi:10.1002/emmm.201202258)

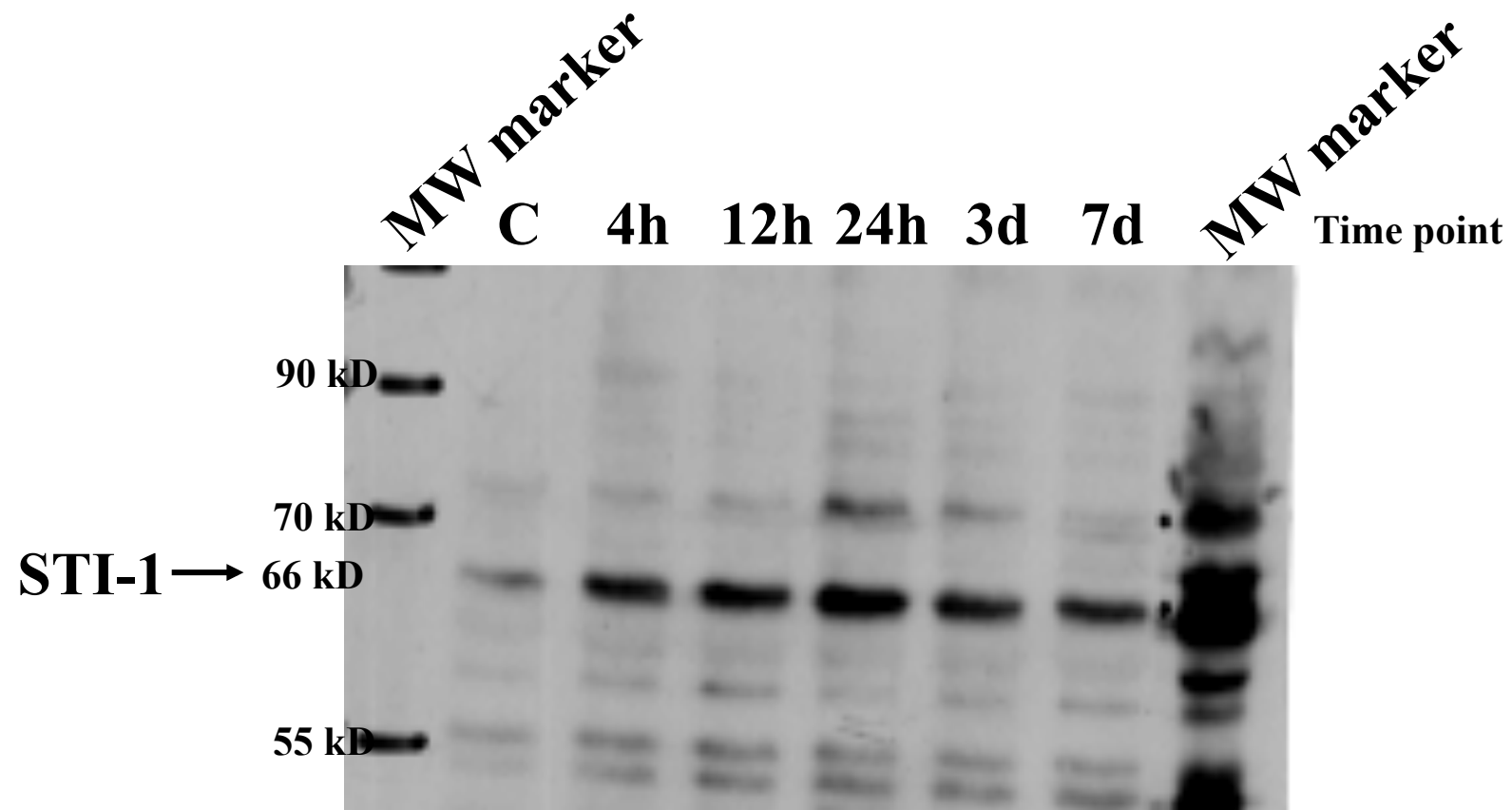

**Figure 1E**

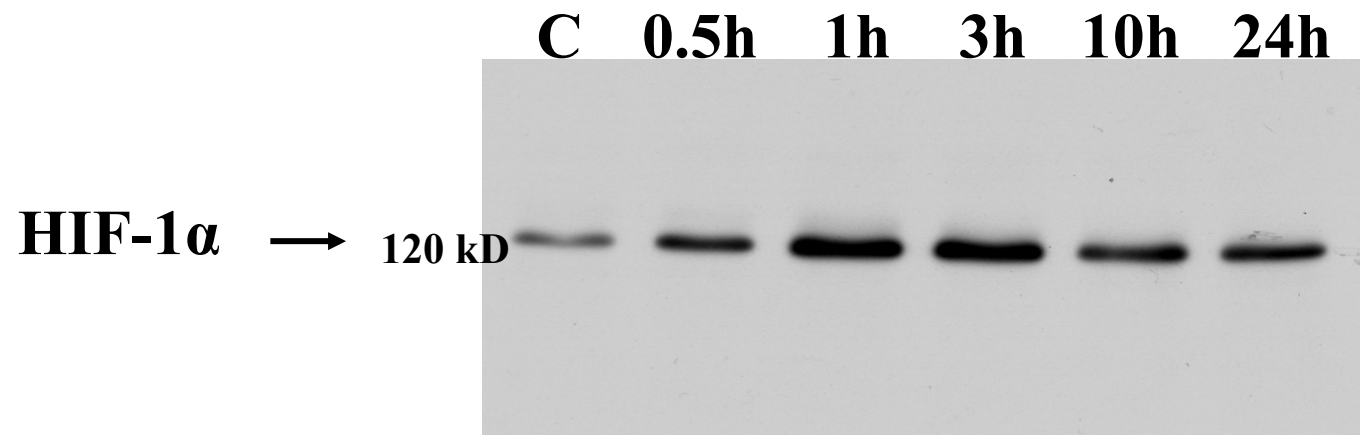

**Figure 2C**

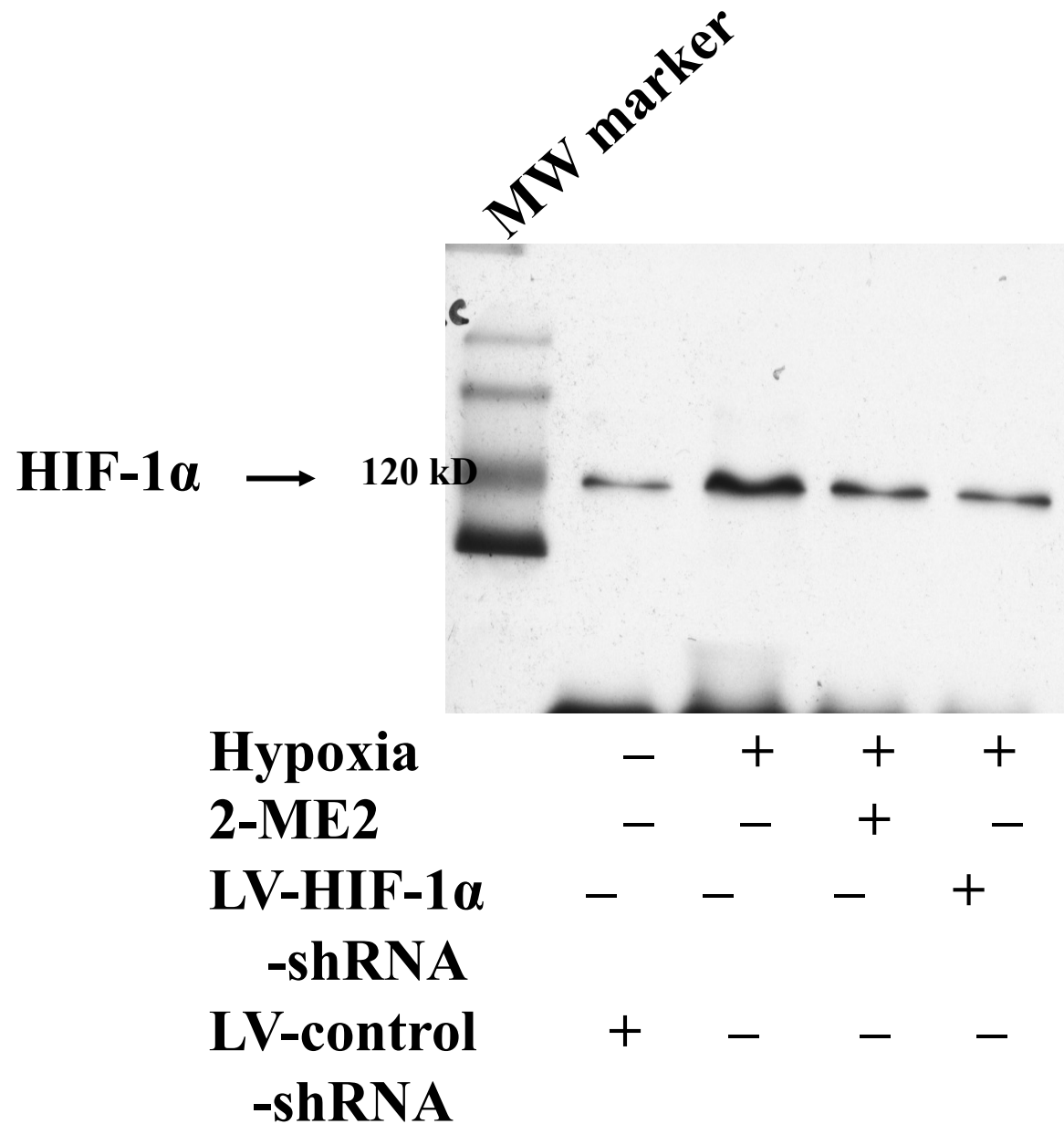

**Figure 3C**

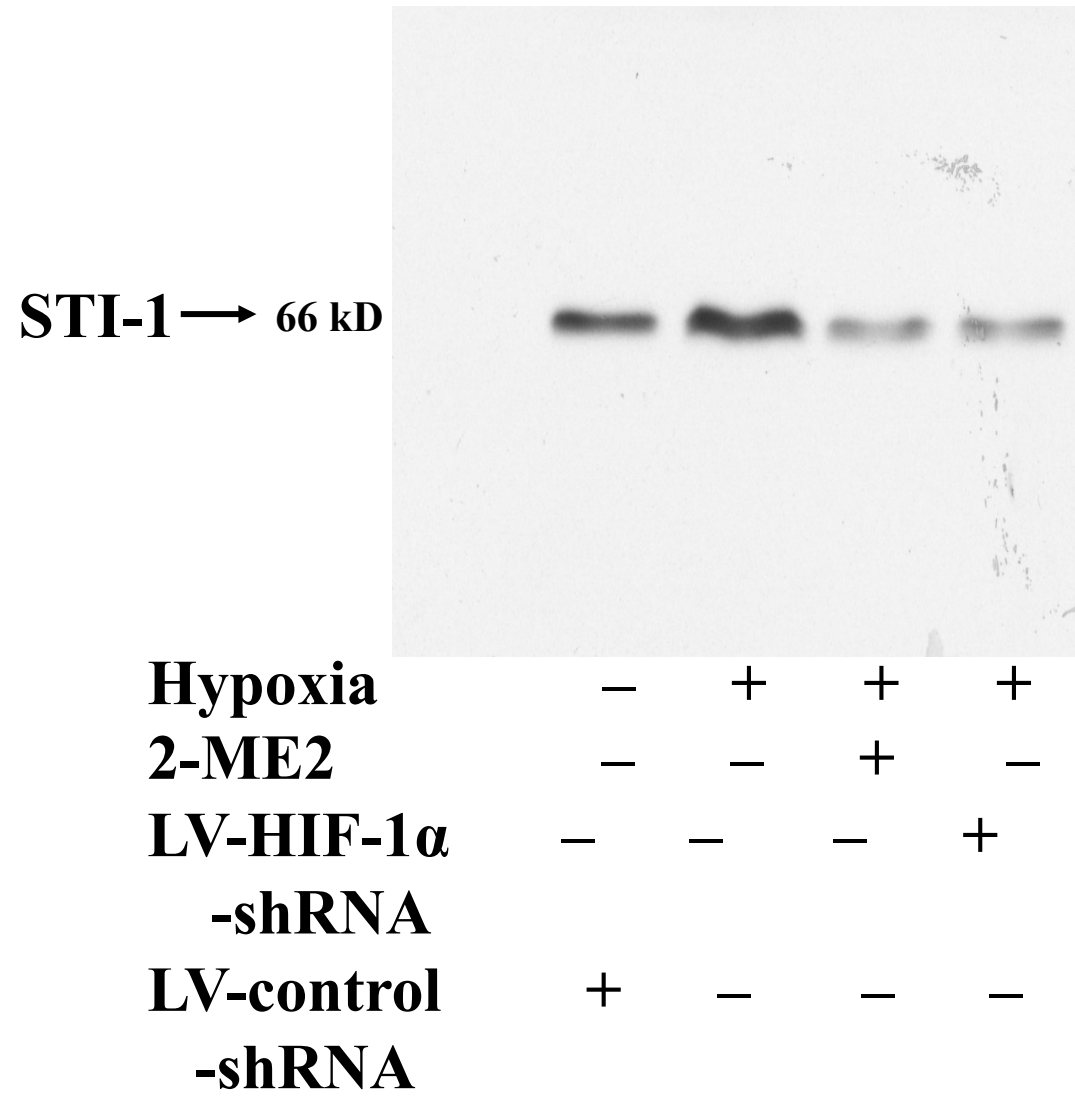

**Figure 3C**

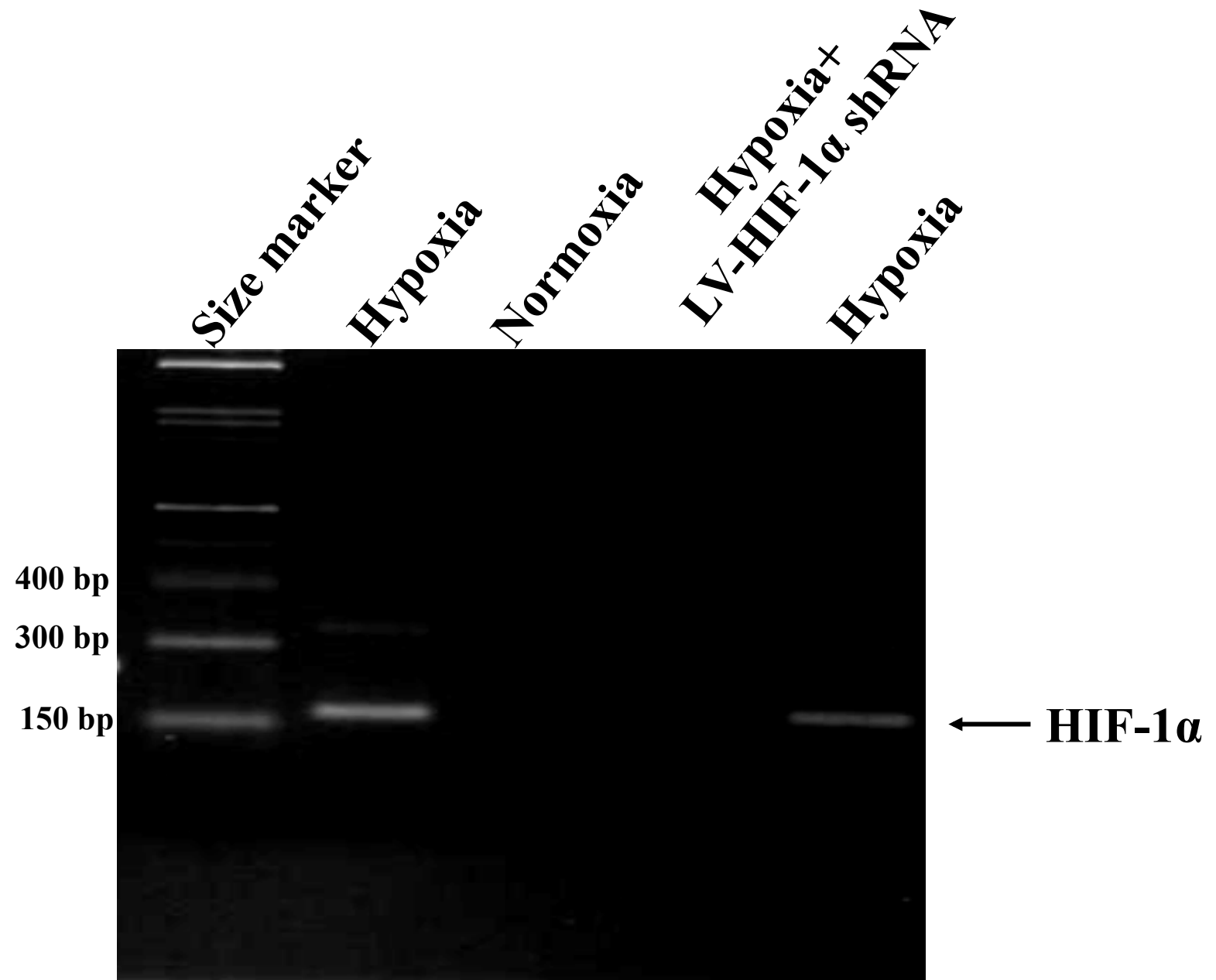

**Figure 3E**

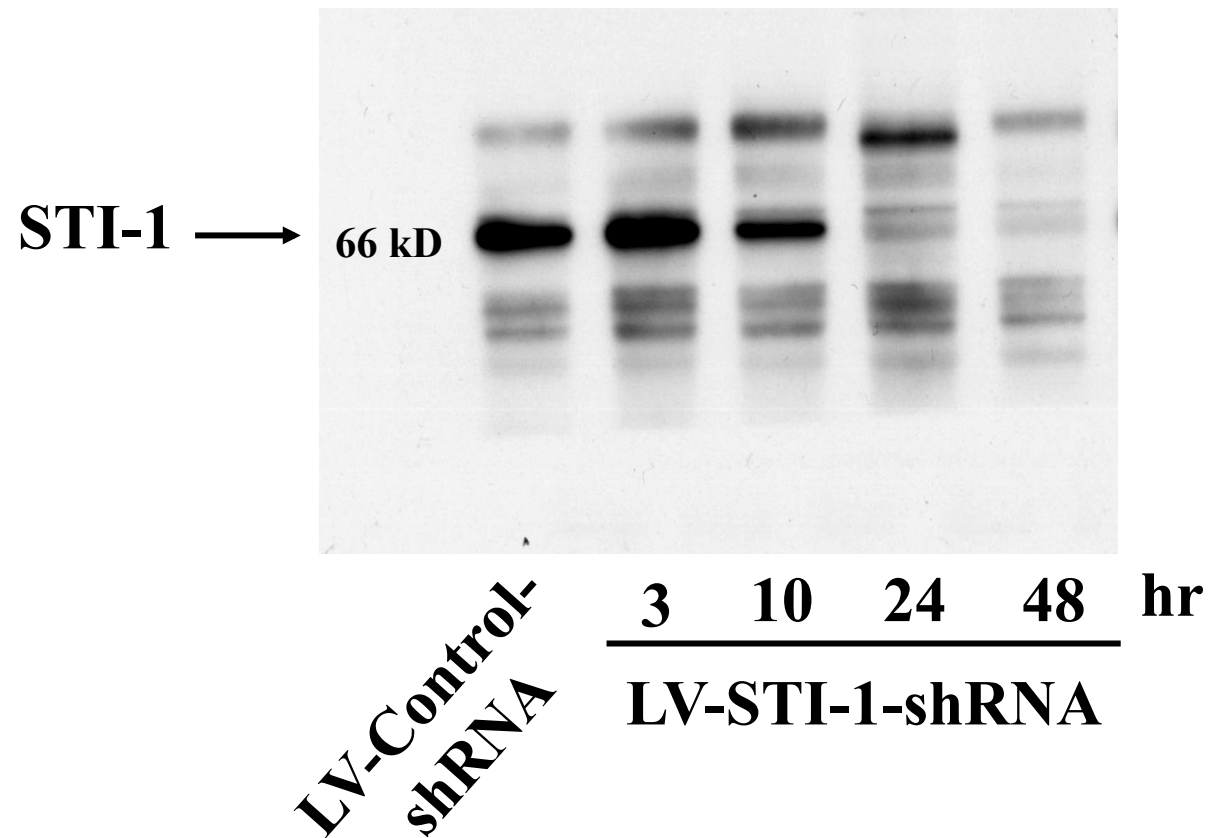

**Figure 7B**

Supplement: Supplementary file 2 [file emmm0005-1227-SD2.pdf]
